# A grand unified model for liganded gold clusters

**DOI:** 10.1038/ncomms13574

**Published:** 2016-12-02

**Authors:** Wen Wu Xu, Beien Zhu, Xiao Cheng Zeng, Yi Gao

**Affiliations:** 1 Division of Interfacial Water and Key Laboratory of Interfacial Physics and Technology, Shanghai Institute of Applied Physics, Chinese Academy of Sciences, Shanghai, 201800 China; 2 Department of Chemistry, University of Nebraska-Lincoln, Lincoln, Nebraska 68588, USA; 3 Collaborative Innovation Center of Chemistry for Energy Materials, University of Science and Technology of China, Hefei, Anhui 230026, China; 4 Shanghai Science Research Center, Chinese Academy of Sciences, Shanghai 201204, China

## Abstract

A grand unified model (GUM) is developed to achieve fundamental understanding of rich structures of all 71 liganded gold clusters reported to date. Inspired by the quark model by which composite particles (for example, protons and neutrons) are formed by combining three quarks (or flavours), here gold atoms are assigned three ‘flavours' (namely, bottom, middle and top) to represent three possible valence states. The ‘composite particles' in GUM are categorized into two groups: variants of triangular elementary block Au_3_(2*e*) and tetrahedral elementary block Au_4_(2*e*), all satisfying the duet rule (2*e*) of the valence shell, akin to the octet rule in general chemistry. The elementary blocks, when packed together, form the cores of liganded gold clusters. With the GUM, structures of 71 liganded gold clusters and their growth mechanism can be deciphered altogether. Although GUM is a predictive heuristic and may not be necessarily reflective of the actual electronic structure, several highly stable liganded gold clusters are predicted, thereby offering GUM-guided synthesis of liganded gold clusters by design.

## Abstract (web summary)

There is no one theoretical model that can explain the stability of all known liganded gold clusters. Here, the authors present a grand unified model, inspired by the quark model of particle physics, which describes gold clusters as combinations of stable triangular Au_3_(2*e*) and tetragonal Au_4_(2*e*) ‘composite particles' built from gold atom ‘elementary particles'.

## Graphical abstract

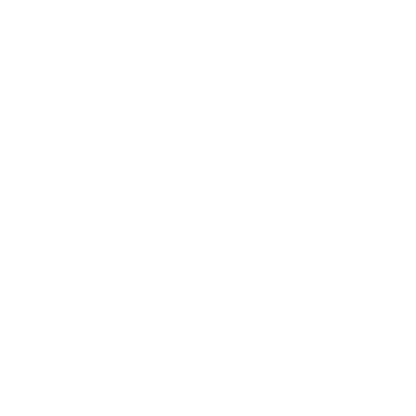

Liganded gold clusters have attracted intensive interest over the past 10 years owing to their broad and practical applications in catalysis^1^, electrochemistry^2^, quantum electronics^3^ and biomedicine^4^. A grand challenge to scientists in this field, however, is the precise determination of atomic structures of liganded gold clusters. To date, atomic structures of tens of liganded gold clusters have been determined via X-ray crystallography^5^,^6^,^7^,^8^,^9^,^10^,^11^,^12^,^13^,^14^,^15^,^16^,^17^,^18^,^19^,^20^,^21^,^22^,^23^,^24^,^25^,^26^,^27^,^28^,^29^,^30^,^31^,^32^,^33^,^34^,^35^,^36^,^37^,^38^,^39^,^40^,^41^,^42^,^43^,^44^,^45^,^46^,^47^,^48^,^49^,^50^,^51^,^52^,^53^,^54^,^55^,^56^,^57^,^58^,^59^,^60^,^61^,^62^,^63^. Nevertheless, these revelations appear serendipitous as the structural determination largely hinges on availability of single crystals for the liganded gold clusters. Although density-functional theory computation has been widely applied to predict the structures of many gold clusters^64^,^65^,^66^,^67^,^68^,^69^,^70^,^71^,^72^,^73^,^74^,^75^,^76^,^77^,^78^, ultimate confirmation still requires X-ray crystallography measurements. Theoretical efforts have also been made in the past for more general unification models to comprehend stabilities of liganded gold clusters with apparently very different and seemingly unrelated complex structures. For example, Wade–Mingos counting rules^79^,^80^ can provide a simple rationale of various shapes of ‘electron-deficient' polyhedral clusters in terms of the number of skeletal electron pairs, particularly for borane and carborane clusters. However, few gold clusters can be rationalized by the Wade–Mingos counting rules^81^, especially for the gold clusters with a high number of interstitial atoms, whose structures have been precisely resolved^60^,^61^. The superatom complex (SAC) model proposed by Walter *et al*.^82^ suggests that the high stability of several spherical-like ligand-protected gold clusters^26^,^55^,^60^ is due largely to the strong electron shell closures, an important concept that stems from the jellium model^83^. The total 16 electronic shell-closing ligand-protected gold clusters (all from previous experiments) are summarized in Supplementary Table 1. Cheng *et al*. developed the super valence bond (SVB) model^84^ to explain the electronic stability of non-spherical shells of Au_38_(SR)_24_ (ref. 53). They suggest that the bi-icosahedral Au_23_^(+9)^ core of Au_38_(SR)_24_ can be viewed as a superatomic molecule. Later, the superatom network (SAN)^66^ model, coupled with the adaptive natural density partitioning analysis^85^, have been also invoked by Cheng *et al*. to explain the high stability of certain low-symmetry ligand-protected gold clusters. A key notion in the SAN model is that the core of Au nanoclusters can be viewed as a network of *n*-centered two-electron (*n*=2–6) superatoms. A tetrahedral unit with two valence electrons has also been identified by Jin and co-workers^58^ through an account of the number of valence electrons and tetrahedral units in a serial of structurally resolved double-helical gold clusters (Au_28_(SR)_20_, Au_36_(SR)_24_, Au_44_(SR)_28_ and Au_52_(SR)_32_)^44^,^49^,^56^,^58^. Recently, the Borromean-ring diagrams for the Au_25_(SR)_18_ (refs 42, 43), Au_38_(SR)_24_ (ref. 53) and Au_102_(SR)_44_ (ref. 60) clusters have been proposed by Pradeep, Whetten and co-workers^86^ to explain high stabilities of these clusters. All the theoretical models developed thus far are mainly to address stabilities of a subset of gold nanoclusters, rather than the entire set of 71 reported liganded gold clusters. As such, exceptional cases to these independent models abound. Hence, a grand unified model that can go beyond these previously developed models (SAC, SVB, SAN and so on) for understanding stabilities of all ligand-protected gold clusters is called for. Here we present a grand unified model (GUM) that can offer a universal description of the structures of diverse liganded gold clusters.

In this communication, the triangular elementary block Au_3_(2*e*) and tetrahedral elementary block Au_4_(2*e*) are identified to describe the stabilities of 71 liganded gold nanoclusters (Supplementary Table 2) reported up to date. On the basis of the GUM, deeper insights into structure evolution of the liganded gold nanoclusters can be obtained, namely, the structure evolution of the gold core cannot be viewed simply as addition of Au atoms, but rather as seamless packing of the elementary blocks. In addition, several stable liganded gold clusters are predicted.

## Results

### GUM development and quark model analogy

**Figure 1:**
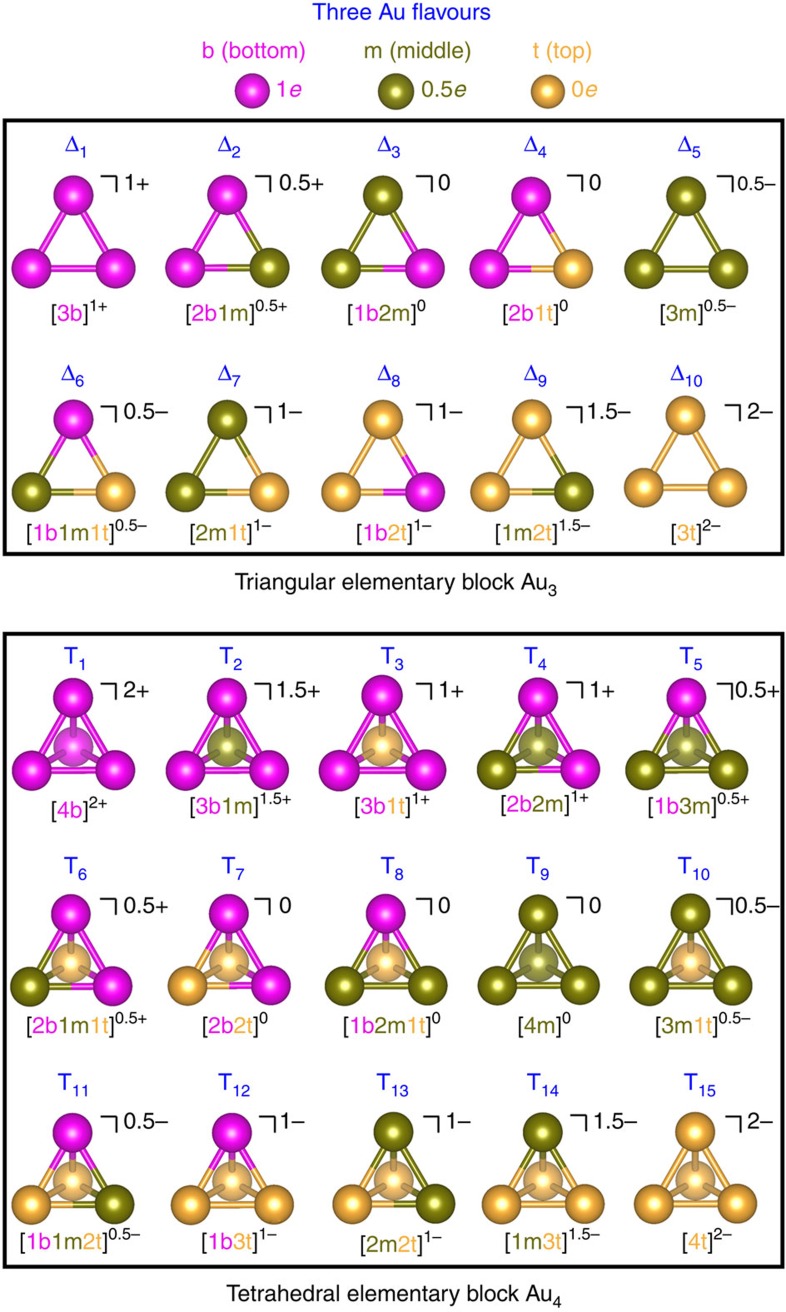
Valence states of the triangular and tetrahedral elementary blocks. Ten variants (Δ_1_–Δ_10_) of valence states for the triangular elementary block Au_3_ and 15 variants (T_1_–T_15_) of valence states for the tetrahedral elementary block Au_4_ due to constituent Au atoms having three possible flavours (b for bottom flavour, m for middle flavour and t for top flavour), and the requirement of duet rule (that is, having 2*e* valence electrons). Colour code of Au atom: magenta (b), dark yellow (m) and yellow (t).

The development of the GUM is based on detailed analysis of the structures of all 71 liganded gold clusters (Supplementary Table 2) either determined from previous experiments (54 crystallized structures) or predicted from density-functional theory computation (17 structures) over the past three decades. The scheme of grand conceptual unification of the diverse structures of these 71 liganded gold clusters is motivated from the quark model in particle physics wherein six types of quarks, known as flavours, are conceptualized as a unification scheme for composite particles, such as protons and neutrons, and exotic hadrons, in terms of their valence quarks. For instance, it is known that protons, neutrons alike are not elementary but are viewed as bound states of the elementary valence quarks and antiquarks. All quarks are characterized by a set of quantum numbers, such as fractional electric charge of ±2/3 or ±1/3. In an analogous fashion, here, we assign a gold atom as the ‘elementary particle' but with one of three ‘flavours' due to its three possible valence states, that is, 1*e*, 0.5*e* and 0*e*. The three flavours are named as bottom, middle and top, respectively. Through close inspection of the 71 known clusters, we identify two ‘composite particles', namely, the triangular Au_3_ and tetrahedral Au_4_ elementary blocks, in analogy to the protons and tetraquarks, respectively. We find that both elementary blocks satisfy the duet rule, that is, the high tendency of having two electrons in the valence shell. As a result, depending on the flavour of each constituent gold atom, the triangular elementary block can exhibit in total 10 variants of valence states (named as Δ_1_–Δ_10_), whereas the tetrahedral elementary block can exhibit in total 15 variants of valence states (named as T_1_–T_15_) (Fig. 1). We show that for all 71 liganded gold clusters, once the outer ligands are effectively detached from the inner Au cores (see below), the resulting Au cores are universally packed by the elementary blocks. Hence, the stabilities of the liganded gold clusters are due to the high stability of each individual elementary block.

### Duet rule

Note that the duet rule elucidated here is akin to the textbook octet rule, a well-known and the first chemical rule of thumb in general chemistry. The octet rule is a valence-electron counting rule for the explanation or prediction of electronic structure and chemical bonding of molecules made of main-group elements. The other two valence-electron counting rules (that is, the second and third rules of thumb), namely, the 18-electron rule and Wade's rule, are newer chemical rules of thumb for understanding chemical structures of organometallics and polyhedral cluster compounds, respectively. Below, we report that in conjunction with the GUM, the duet rule of the valence shell for the elementary blocks can be treated as the fourth rule of thumb for understanding diverse liganded gold clusters.

### Evidences on stabilities of the elementary blocks

**Figure 2:**
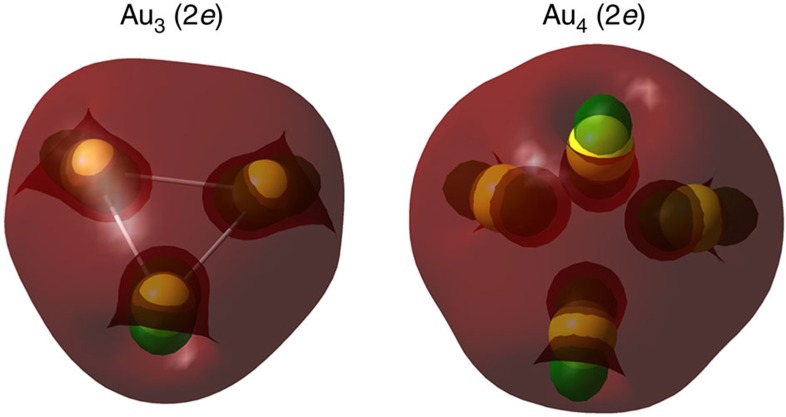
The computed delocalized occupied orbitals (1S^2^) of Au_3_(2*e*) (left) and Au_4_(2*e*) (right). Colour code: Au—yellow.

In GUM, both elementary blocks triangular Au_3_ and tetrahedral Au_4_ entail only two valence electrons [Au_3_(2*e*) and Au_4_(2*e*)], thereby both having strong electron shell closures. As shown in Fig. 2, the two valence electrons are delocalized in the shell-closing elementary blocks Au_3_(2*e*) and Au_4_(2*e*), consistent with the SAC model^82^. Moreover, from an experimental perspective, the Au_3_ core of crystallized [Au_3_(IDipp)_3_]^1+^ [IDipp=1,3-bis(2,6-diisopropylphenyl)imidazol-2-ylidene] (ref. 5) and the Au_4_ core of [Au_4_(PR_3_)_4_]^2+^ (ref. 6) (Supplementary Table 2) are essentially the same as the elementary blocks Au_3_(2*e*) and Au_4_(2*e*), respectively, supporting the high stabilities of the Au_3_(2*e*) and Au_4_(2*e*). The high stabilities of Au_3_(2*e*) and Au_4_(2*e*) structures can be also shown from *ab initio* computation. In Supplementary Table 3, the formation energies of five isoelectronic species, Au_2_(2*e*), Au_3_(2*e*), Au_4_(2*e*), Au_5_(2*e*) and Au_6_(2*e*), are listed. Only the triangular Au_3_(2*e*) and tetrahedral Au_4_(2*e*) exhibit highly negative formation energies, which provides another piece of strong evidence of their high stabilities. In addition, both elementary blocks exhibit larger highest/lowest occupied/unoccupied molecular orbital (HOMO/LUMO) gaps than their isoelectronic counterparts (Supplementary Table 3).

### Electron counting protocols for effective detachment of ligands

**Figure 3:**
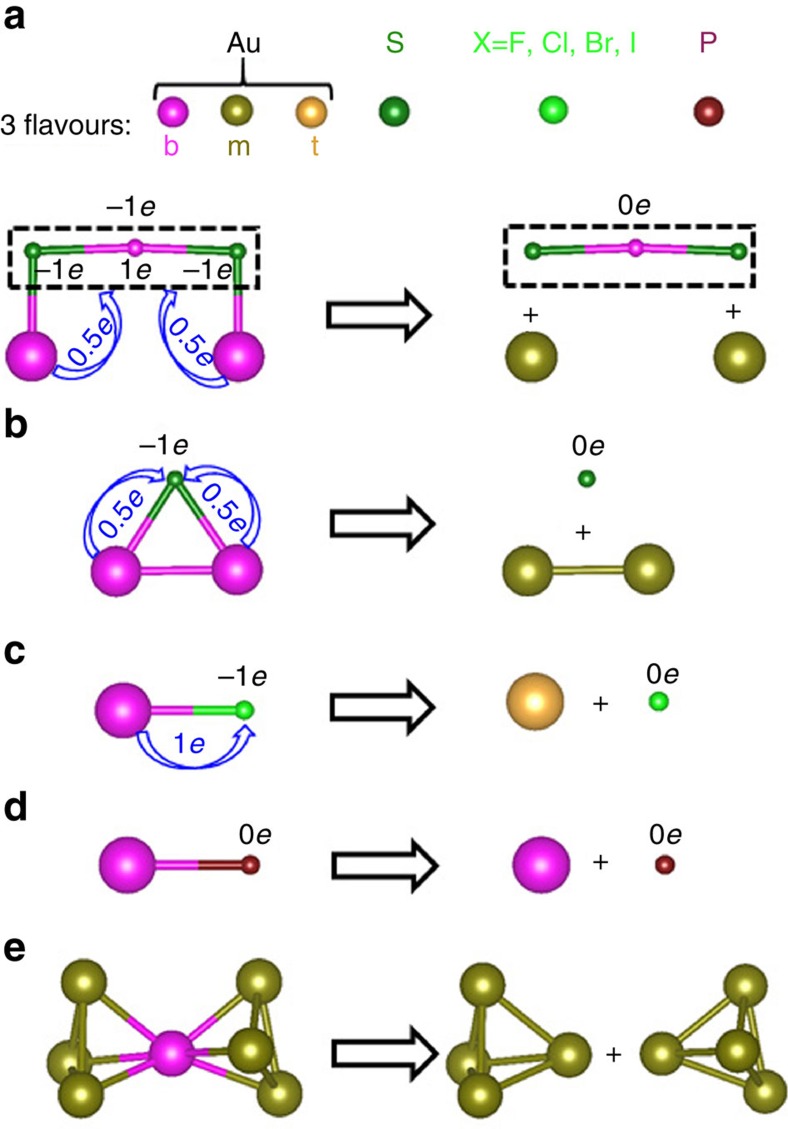
Electron counting protocols for effective detachment of ligands from inner Au core. There are four cases for Au atom bonded with (**a**) gold–thiolate staple motifs, (**b**) SR, (**c**) X (X=F, Cl, Br and I) and (**d**) PR_3_ functional groups; (**e**) effective separation of an Au atom shared by two elementary blocks. The blue arrow denotes charge transfer. Colour code: Au—magenta (bottom flavour), dark yellow (middle flavour) and yellow (top flavour); S—dark green; X—light green; P—wine. The R groups are omitted for clarity.

All ligand-protected Au clusters are composed of an inner Au core and a number of outer ligands. The first step toward developing the GUM is to find effective protocols to detach the protection ligands from the Au core so that the vast complex factors of the outer ligands can be removed while all ligand-protected Au clusters can be reduced to bare Au cores for structural analysis. Note that different elements or functional groups that are directly bonded with an Au atom at the core surface are in different valence-electron states. As such, if one is to focus on the valence state of the inner Au core, equivalent electron counting is required to effectively detach all protection ligands from the Au core. The following electron-counting protocols for effective detachment of different types of ligands can be undertaken (see Fig. 3 for graphical illustrations):

First, each SR group and Au atom embedded in the gold–thiolate staple motifs possess −1*e* and 1*e* valence electron, respectively. To effectively detach the smallest staple motif from the Au core, the net number of valence electrons of the staple motif should be converted to zero. To this end, each of the two Au atoms (on the Au core) bonded with SR is considered to transfer 0.5*e* valence electron to the staple motif. As such, equivalent electron counting for effective detachment of the staple motif is achieved (Fig. 3a). Second, for SR group bonded with two Au atoms on the Au core, again, each of the two Au atoms (on the core) is considered to transfer 0.5*e* valence electron to the SR group so that equivalent electron counting for effective detachment of the SR group is achieved (Fig. 3b). Third, for halogen ligand X (X=F, Cl, Br and I) bonded with a single Au atom on the Au core, each Au atom is considered to transfer 1*e* valence electron to the X atom so that equivalent electron counting for effective detachment of the X atom is achieved (Fig. 3c). Fourth, the phosphine group is known as a weak ligand, thereby possessing 0*e* valence electron. As such, each Au atom bonded with the phosphine group still maintain its original 1*e* valence electron upon detachment with the phosphine group (Fig. 3d).

In summary, depending on the ligands, for example, PR_3_, X, SR or gold–thiolate staple motifs, Au atoms on the Au core can exhibit one of the three flavours: bottom (1*e*), middle (0.5*e*) and top (0*e*) after effective detachment of the protection ligands from the Au core. Note also that when two elementary blocks are fused together via sharing a single Au atom, the shared Au atom contributes 0.5*e* valence electron to each elementary block. As such, the fused elementary blocks can be effectively separated via the protocol shown in Fig. 3e.

### Prototypical liganded clusters

As shown in Supplementary Table 2, once the ligands are effectively detached from the Au cores, the Au cores of all 71 ligand-protected gold clusters can be universally decomposed into a number of the triangular Au_3_ (Δ_1_–Δ_10_) and/or tetrahedral Au_4_ (T_1_–T_15_) elementary blocks. Two prototypical structures are analysed here as two examples while other eight representative structures are either briefly illustrated here or elaborated in the Supplementary Figs 1–8. The remaining cases can be analysed in similar fashion.

**Figure 4:**
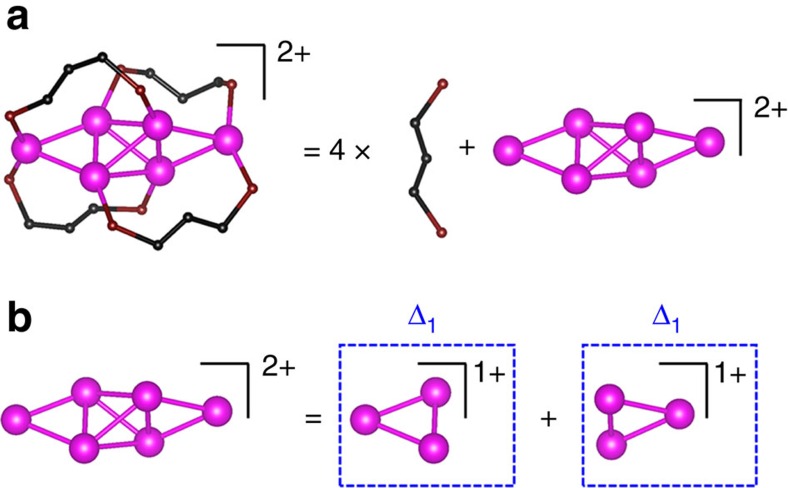
The structural decomposition of [Au_6_(dppp)_4_]^2+^. (**a**) The decomposition of [Au_6_(dppp)_4_]^2+^ cluster into four ligands and an Au core. (**b**) The decomposition of the inner Au core into two triangular Au_3_ elementary blocks, each block being in the Δ_1_ valence state. Colour code: Au—magenta (bottom flavour); C—black; P—wine. The R groups are omitted for clarity.

The first prototype structure we consider is [Au_6_(dppp)_4_]^2+^ (dppp=1,3-Bis(diphenylphosphino)propane)^9^, which is composed of four dppp ligands (each with 0*e* valence electron) and an Au_6_^2+^ core (Fig. 4a). The Au_6_ core consists of two triangular Au_3_ blocks. According to the electron-counting protocol, the six Au atoms of the Au_6_ core are all bonded with phosphine ligands and thus have 1*e* valence electron or the bottom flavour. Two positive charges are equally distributed in the two triangular Au_3_ blocks. Thus, both triangular Au_3_ elementary blocks are in the Δ_1_ valence state (Fig. 4b).

**Figure 5:**
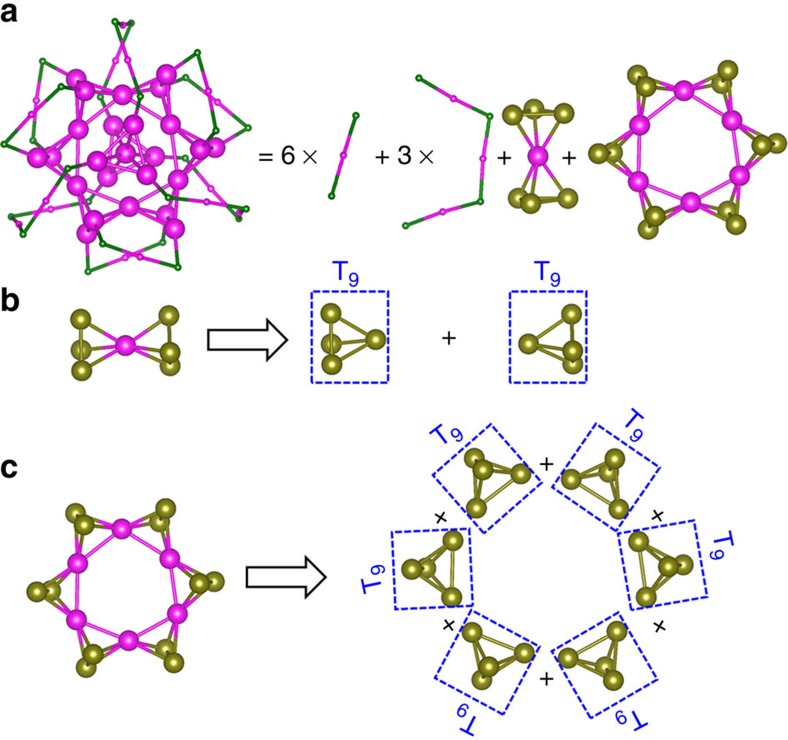
The structural decomposition of Au_40_(SR)_24_. (**a**) The decomposition of Au_40_(SR)_24_ cluster into nine ligands and an Au core with two shells. (**b**) The decomposition of inner shell of the Au core into two fused tetrahedral Au_4_ elementary blocks, each block, when separated, being in the T_9_ valence state. (**c**) The decomposition of the outer shell of the Au core into six fused tetrahedral Au_4_ elementary blocks in a loop, each block, when separated, being in the T_9_ valence state. Colour code: Au—magenta (bottom flavour) and dark yellow (middle flavour); S—dark green. The R groups are omitted for clarity.

The second prototype structure we consider is Au_40_(SR)_24_ (ref. 56). The Au core of Au_40_(SR)_24_ can be viewed as a combination of an Au_7_ and a Kekulé-like Au_18_ structure (Fig. 5a). The Au_7_ core is composed of two tetrahedral Au_4_ blocks, fused together by sharing an Au atom. The latter contributes 0.5*e* valence electron to each tetrahedral Au_4_ block. Other six vertex Au atoms are bonded with the SR group and each contributes 0.5*e* valence electron to the resident tetrahedral Au_4_ block. Thus, each of the four vertex Au atoms in the tetrahedral Au_4_ block has the middle flavour so that each tetrahedral Au_4_ elementary block is in the T_9_ valence state (Fig. 5b). Moreover, the Kekulé-like Au_18_ structure can be viewed as six tetrahedral Au_4_ blocks fused together in a loop with six sharing Au atoms (Fig. 5c). Again, each of the six tetrahedral Au_4_ elementary blocks is in the T_9_ valence state.

Finally, eight other representative clusters are considered. The structure decompositions of eight other representative clusters in terms of elementary blocks as well as their corresponding valence states are shown in Supplementary Figs 1–8. Specifically, the structure decomposition of three largest ligand-protected gold clusters, Au_102_(SR)_44_ (ref. 60), Au_130_(SR)_50_ (ref. 61) and Au_144_(SR)_60_ (ref. 78), are given below. According to the ‘divide-and-protect' formulation^87^,^88^, the Au_102_(SR)_44_ can be written as Au_79_[RS-Au-SR]_19_[RS-Au-SR-Au-SR]_2_. Each of the two Au atoms in the Au_102_(SR)_44_ shared by two [-RS-Au-SR-] staple motifs can be viewed as the Au atom with 0*e* valence electron (that is, top flavour). As such, the Au_79_ core of Au_102_(SR)_44_ is composed of 29 elementary blocks and their corresponding valence states can be described by [5Δ_1_, Δ_3_, 16Δ_5_, 2T_4_, 2T_5_, T_7_ and 2T_9_] (Supplementary Fig. 9), giving rise to 58 valence electrons in total (corresponding to the strong electron shell closure, according to SAC model^82^) for Au_102_(SR)_44_. The Au_105_ core of Au_130_(SR)_50_ is composed of 40 elementary blocks and their corresponding valence states include 5Δ_3_, 11Δ_5_, 4T_4_, 3T_5_ and 17T_9_ (Supplementary Fig. 10), giving rise to 80 valence electrons in total for Au_130_(SR)_50_. The Au_114_ core of Au_144_(SR)_60_ is composed of 42 elementary blocks and their corresponding valence states include 20Δ_5_, 13T_4_, 3T_5_ and 6T_9_ (Supplementary Fig. 11), giving rise to 84 valence electrons in total in Au_144_(SR)_60_.

## Discussion

**Figure 6:**
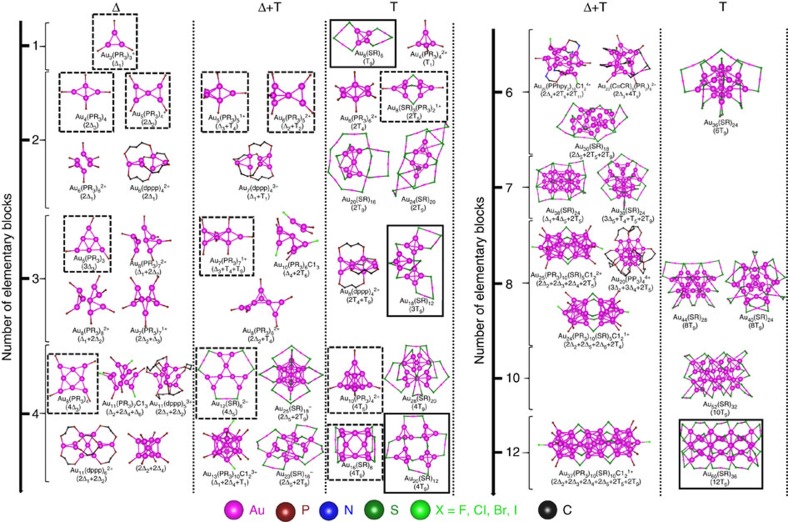
Structure evolution of the Au cores (large magenta spheres) with increasing number of elementary blocks. Colour code: Au—magenta; S—dark green; X—light green; P—wine; C—black; N—blue. The R groups are omitted for clarity. The dotted and solid squares denote the newly and previously predicted structures, respectively. Others are crystallized structures.

In general, the inner Au cores of all the ligand-protected gold clusters are composed of elementary blocks (Supplementary Table 2 and Fig. 6), each having two valence electrons (Fig. 1), whereas the outer ligands with 0*e* valence electron provide the geometry constraint and arrangement of valence electrons in each elementary block to satisfy the duet rule. In Supplementary Fig. 12, we show several possible valence states for two fused tetrahedral Au_4_ blocks (Au_7_) protected by either SR or PR_3_ groups in various liganded gold clusters. Our analysis indicates that the fused tetrahedral Au_4_ elementary blocks in the four predicted stable clusters, Au_10_(SR)_6_, [Au_9_(SR)_4_(PR_3_)_2_]^1+^, [Au_8_(SR)_2_(PR_3_)_4_]^2+^ and [Au_7_(PR_3_)_6_]^3+^, are in the 2T_9_, 2T_5_, 2T_4_ and 2T_2_ valence states, respectively, whereas the constructed Au_8_(SR)_2_(PR_3_)_4_ and Au_7_(PR_3_)_6_ clusters are expected to be less stable due to their violation of the duet rule. Note that a recent experimental investigation of Au_25_(SR)_18_ in its three oxidation states, that is, Au_25_(SR)_18_^−1/0/+1^, provides more compiling evidence on the effect of violation of the duet rule (or deviation from the strong electron shell closure) to the symmetry of the Au_13_ core and stability of the cluster^89^. The obtained three crystalline structures in the related three oxidation states demonstrate that the structural distortion (in the Au core in particular) increases with the decreased superatomic valence from 1S^2^1P^6^ to 1S^2^1P^4^ as the Au_25_(SR)_18_^−1^ cluster has the eight-electron shell-closing configuration^82^ 1S^2^1P^6^, and thus the highest thermal stability. The other two oxidation states, Au_25_(SR)_18_^0/+1^, are less stable due to the incomplete 1P superatomic orbital. Likewise, the structural distortion observed in the two oxidation states, Au_25_(SR)_18_^0/+1^, can be understood based on GUM. The Au_13_ core of Au_25_(SR)_18_^−1^ can be decomposed into four elementary blocks and their corresponding valence states can be described as [2Δ_5_ and 2T_9_] (Supplementary Table 2). Clearly, the number of valence electrons in both oxidation states, Au_25_(SR)_18_^0/+1^, dissatisfies the duet rule. As a consequence, lower thermal stability and larger structural distortion in the highly symmetric Au_13_ core are expected, consistent with the experimental evidence^89^.

The GUM does not only offer a universal structural characterization of all 71 liganded gold clusters, but it also provides deeper insights into structure evolution of the Au clusters. In Fig. 6, a structure evolution map for the Au cores with increasing number of elementary blocks is presented. Indeed, the structure evolution of the Au cores can be understood through various routes of packing the elementary blocks. For example, two elementary blocks with T_9_ valence state can yield either the Au_8_ core of Au_24_(SR)_20_ (ref. 39) via direct packing or the Au_7_ core of Au_20_(SR)_16_ (ref. 33) via sharing a vertex Au atom. Two elementary blocks with T_4_ valence states can give rise to the Au_6_ core of [Au_6_(PR_3_)_6_]^2+^ (ref. 7) through sharing one common edge. With gradually increasing the number of elementary blocks, larger Au core structures can be formed while adjusting the overall charge of the liganded gold clusters to meet the duet rule. In summary, the structure evolution of the Au core cannot be viewed simply as addition of Au atoms (Supplementary Fig. 13), but rather as seamless packing of the elementary blocks while obeying the duet rule.

In addition to structure unification, the GUM can be utilized for predicting new structures of liganded gold clusters. From the structure evolution map shown in Fig. 6, one can see that there are many vacant spaces, suggesting many missing liganded gold clusters yet to be synthesized. We construct a series of ligand-protected gold clusters (shown in dotted squares in Fig. 6) on the basis of GUM to fill some vacant spaces in Fig. 6. The elementary blocks and their valence states for the Au cores of all constructed clusters are depicted in Supplementary Fig. 14. These clusters exhibit large computed HOMO–LUMO gaps (Supplementary Table 4), suggesting potentially high chemical stabilities.

**Figure 7:**
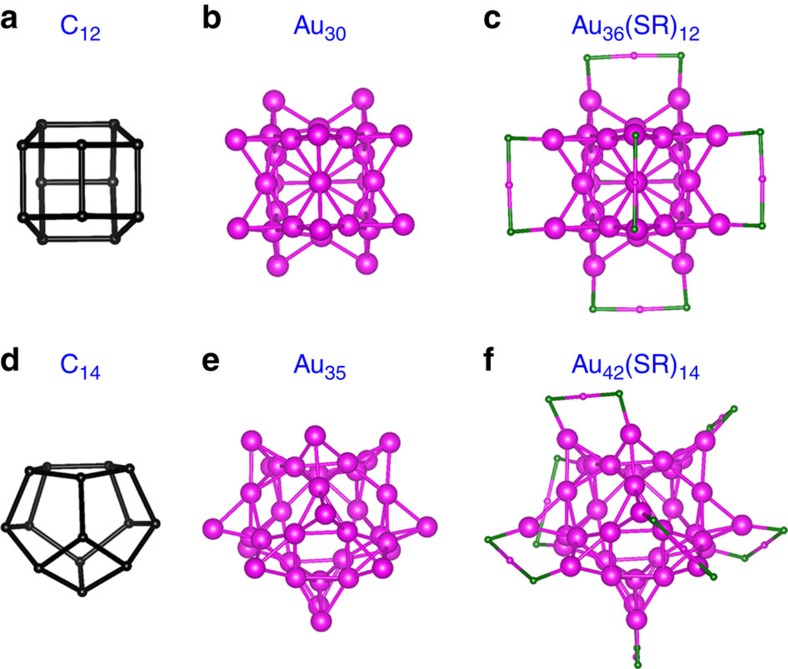
Two predicted Au_36_(SR)_12_ and Au_42_(SR)_14_ clusters based on GUM using C_12_ and C_14_ fullerenes as templates. Structures of a C_12_ fullerene (**a**), Au_30_ core (**b**) and Au_36_(SR)_12_ cluster (**c**), as well as C_14_ fullerene (**d**), Au_35_ core (**e**) and Au_42_(SR)_14_ cluster (**f**). Colour code: Au—magenta; S—dark green; C—black. The R groups are omitted for clarity.

In particular, a class of hitherto unreported ligand-protected hollow Au clusters are presented here. For example, Au_36_(SR)_12_ can be constructed by using the C_12_ fullerene as a template. C_12_ fullerene exhibits eight polygons: four quadrilaterals and four pentagons (Fig. 7a). Replacing all C atoms of the C_12_ fullerene with 12 fused tetrahedral Au_4_ gives rise to the Au_30_ core (Fig. 7b) of Au_36_(SR)_12_, followed by adding [-RS-Au-SR-] staple motifs on the unfused Au atoms to build the complete Au_36_(SR)_12_ cluster (Fig. 7c). The Au_30_ hollow cage in Au_36_(SR)_12_ is composed of 12 fused elementary blocks all at the T_9_ valence state. The Au_36_(SR)_12_ cluster exhibits a large computed HOMO–LUMO gap of 2.20 eV and has no imaginary vibrational frequencies. An *ab initio* molecular dynamics simulation of the Au_36_(SR)_12_ cluster at 355 K for 10 ps suggests high thermal stability of the Au_36_(SR)_12_ cluster (Supplementary Fig. 15 and Supplementary Methods). Figure 7f shows another example of ligand-protected hollow Au cluster, namely, Au_42_(SR)_14_, constructed by using the C_14_ fullerene (Fig. 7d) as a template. The Au_35_ hollow cage (Fig. 7e) in Au_42_(SR)_14_ is composed of 14 fused elementary blocks all at the T_9_ valence state. The computed HOMO–LUMO gap of Au_42_(SR)_14_ is 2.00 eV, suggesting high chemical stability. Interestingly, the Au_36_(SR)_12_ and Au_42_(SR)_14_ can be rewritten as Au_30_[Au(SR)_2_]_6_ and Au_35_[Au(SR)_2_]_7_, respectively, consistent with the ‘divide-and-protect' formulation^87^,^88^.

In conclusion, a grand unified model that can incorporate previously developed independent models (SAC, SVB, SAN and so on.) is developed to address stabilities of all ligand-protected gold clusters. On the basis of the GUM, all 71 liganded gold nanoclusters can be decomposed into several elementary blocks of triangular Au_3_(2*e*) and tetrahedral Au_4_(2*e*). Although GUM is a predictive heuristic and may not be necessarily reflective of the actual electronic structure, a series of highly stable liganded gold clusters are predicted, which provides a guide to synthesizing new ligand-protected gold clusters. Hence, the GUM can offer not only new insights into the packing and structure evolution of the 71 liganded gold clusters known as of today, but also a systematic route toward rational design and characterization of liganded metal clusters to inspire future experimental synthesis.

### Data availability

The authors declare that the data supporting the findings of this study are available within the article and its Supplementary Information files, and all relevant data are available from the authors.

## Additional information

**How to cite this article:** Xu, W. W. *et al*. A grand unified model for liganded gold clusters. *Nat. Commun.* **7,** 13574 doi: 10.1038/ncomms13574 (2016).

**Publisher's note**: Springer Nature remains neutral with regard to jurisdictional claims in published maps and institutional affiliations.

## Supplementary Material

Supplementary Information Supplementary Figures 1-15, Supplementary Tables 1-4, Supplementary Methods and Supplementary References.
