# Supplementary material for: A grand unified model for liganded gold clusters
Source: Nat Commun. 2016 Dec 2;7:13574. doi: 10.1038/ncomms13574 (PMC5146290; doi:10.1038/ncomms13574)
Supplement: Supplementary Information — Supplementary Figures 1-15, Supplementary Tables 1-4, Supplementary Methods and Supplementary References. [file ncomms13574-s1.pdf]

Supplementary Figures:

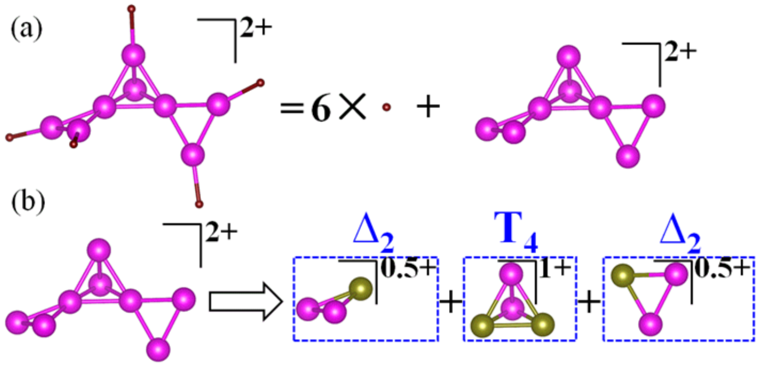

Supplementary Figure 1. Structure decomposition of (a) the $[Au_8(PR_3)_6]^{2+}$[12] cluster and (b) the Au core. Color code: Au – magenta (bottom flavor) and dark yellow (middle flavor); P – wine. The R groups are omitted for clarity.

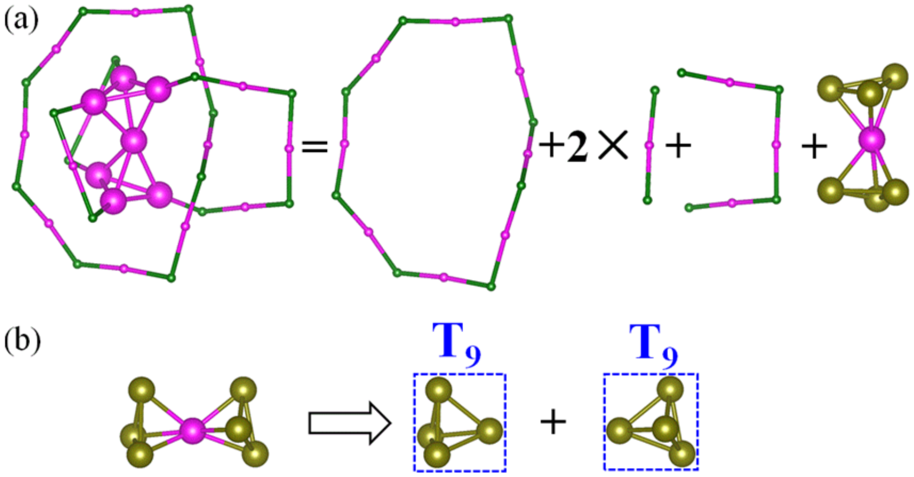

Supplementary Figure 2. Structure decomposition of (a) the $Au_{20}(SR)_{16}$[29] cluster and (b) the Au core. Color code: Au – magenta (bottom flavor) and dark yellow (middle flavor); S – dark green. The R groups are omitted for clarity.

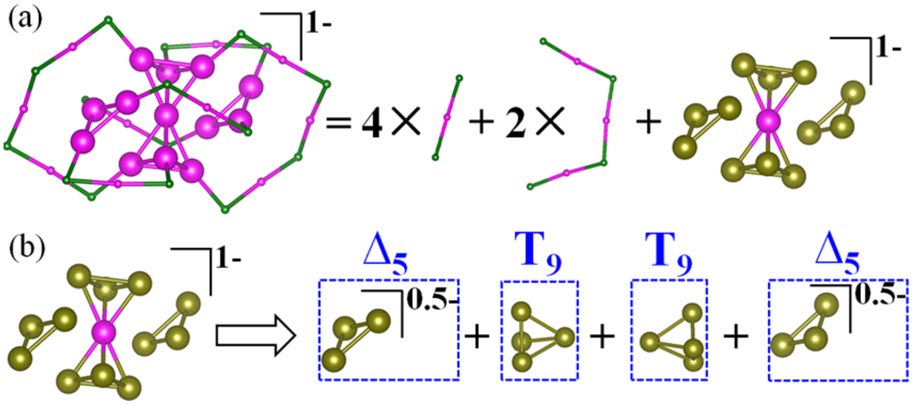

Supplementary Figure 3. Structure decomposition of (a) the $[Au_{23}(SR)_{16}]^-$ [32] cluster and (b) the Au core. Color code: Au – magenta (bottom flavor) and dark yellow (middle flavor); S – dark green. The R groups are omitted for clarity.

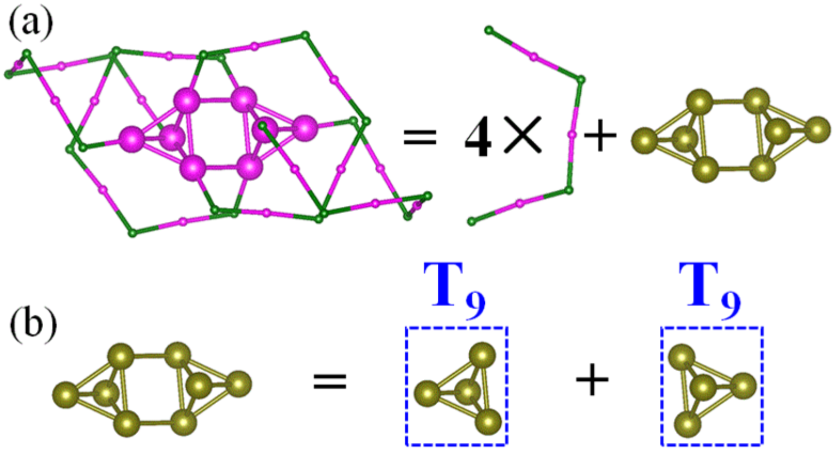

Supplementary Figure 4. Structure decomposition of (a) the $Au_{24}(SR)_{20}$ [35] cluster and (b) the Au core. Color code: Au – magenta (bottom flavor); S – dark green. The R groups are omitted for clarity.

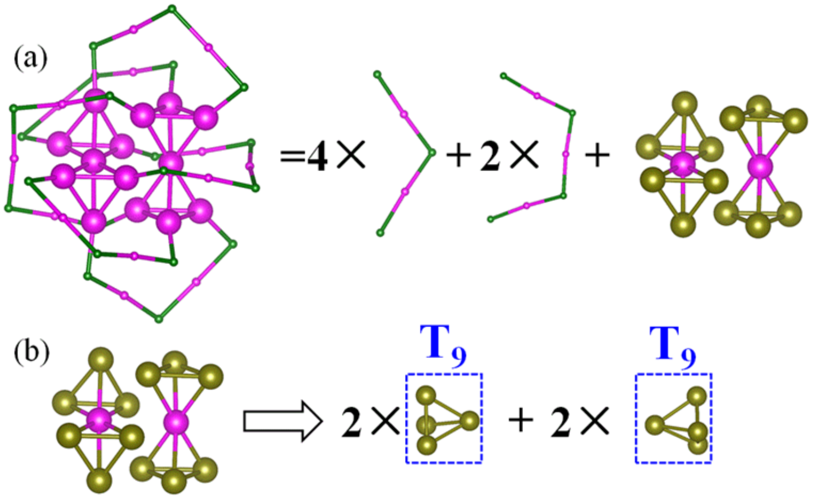

<u>Supplementary</u> Figure 5. Structure decomposition of (a) the $Au_{28}(SR)_{20}$ [40] cluster and (b) the Au core. Color code: Au – magenta (bottom flavor) and dark yellow (middle flavor); S – dark green. The R groups are omitted for clarity.

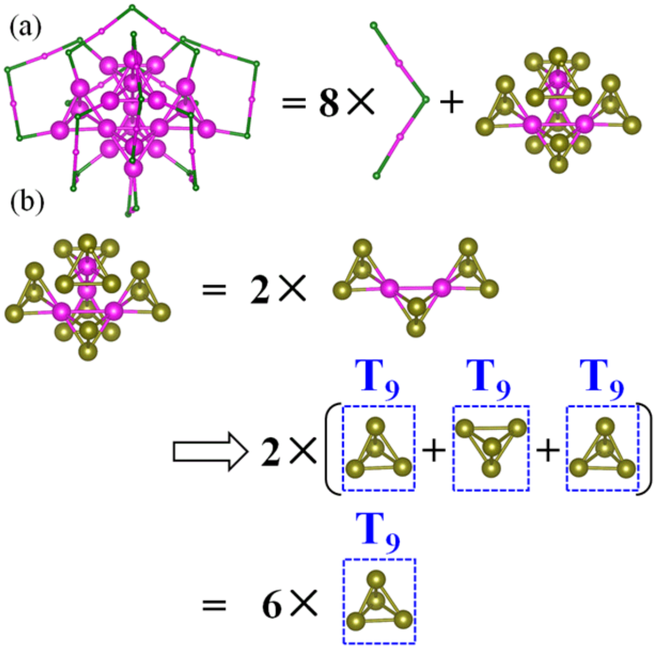

<u>Supplementary</u> Figure 6. Structure decomposition of (a) the $Au_{36}(SR)_{24}$ [45] cluster and (b) the Au core. Color code: Au – magenta (bottom flavor) and dark yellow (middle flavor); S – dark green. The R groups are omitted for clarity.

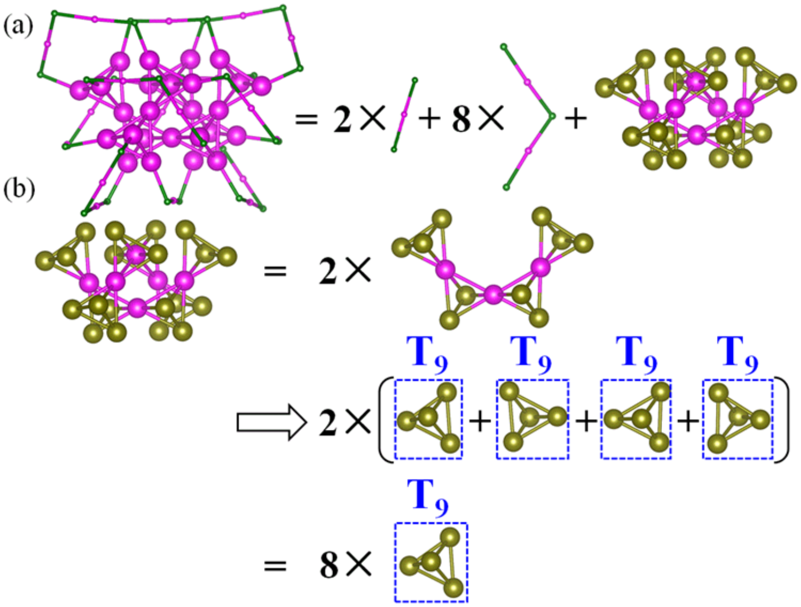

Supplementary Figure 7. Structure decomposition of (a) the $Au_{44}(SR)_{28}$ [53] cluster and (b) the Au core. Color code: Au – magenta (bottom flavor) and dark yellow (middle flavor); S – dark green. The R groups are omitted for clarity.

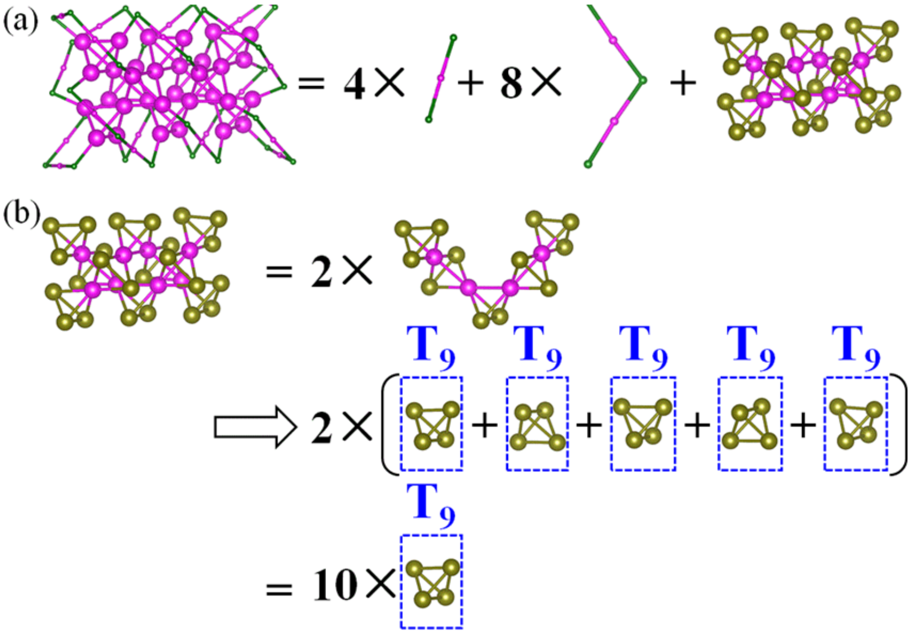

Supplementary Figure 8. Structure decomposition of (a) the $Au_{52}(SR)_{32}$ [51] cluster and (b) the Au core. Color code: Au – magenta (bottom flavor) and dark yellow (middle flavor); S – dark green. The R groups are omitted for clarity.

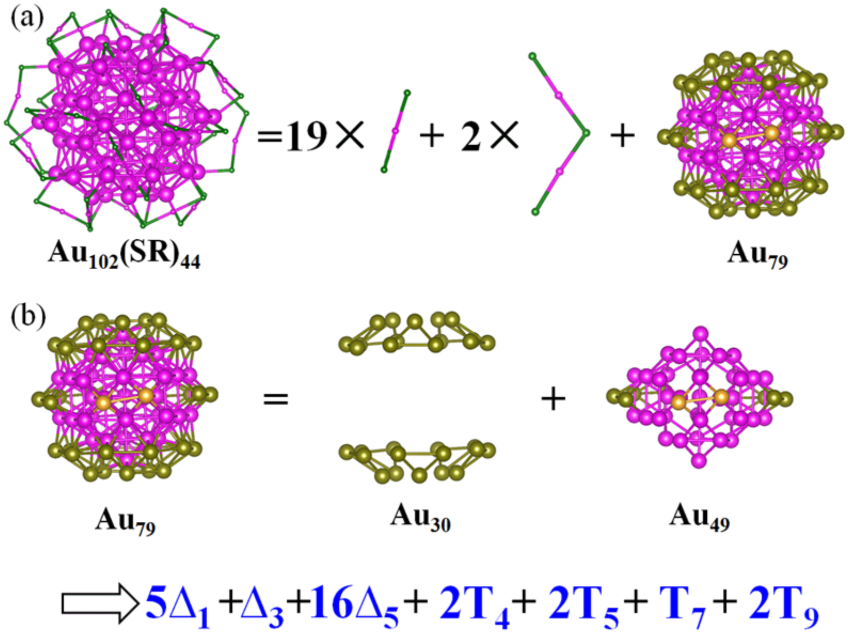

<u>Supplementary</u> Figure 9. Structure decomposition of (a) the $Au_{102}(SR)_{44}$ cluster and (b) the Au_{79} core. Color code: Au – magenta (bottom flavor), dark yellow (middle flavor), and yellow (top flavor); S – dark green. The R groups are omitted for clarity.

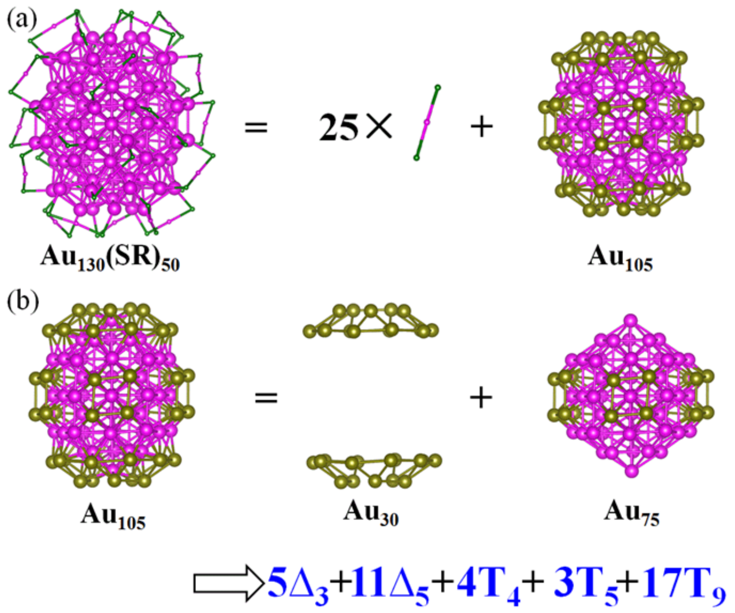

<u>Supplementary</u> Figure 10. Structure decomposition of (a) the $Au_{130}(SR)_{50}$ cluster and (b) the Au_{105} core. Color code: Au – magenta (bottom flavor) and dark yellow (middle flavor); S – dark green. The R groups are omitted for clarity.

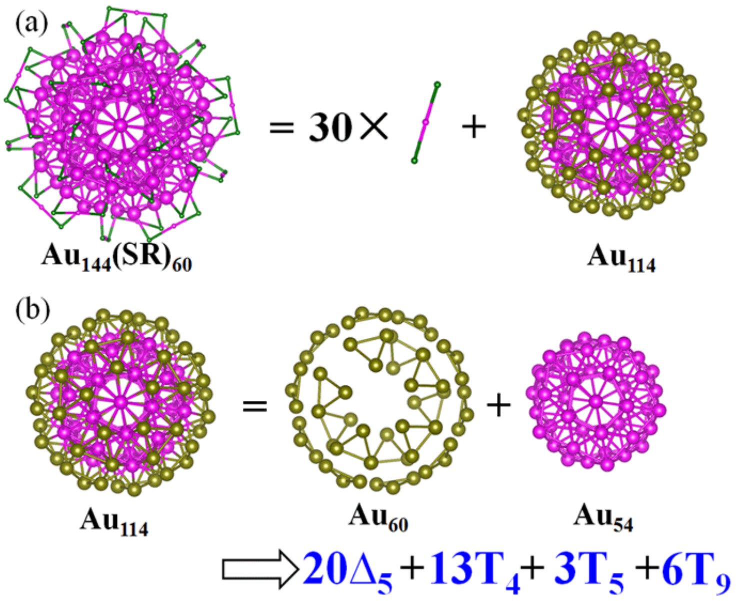

Supplementary Figure 11. Structure decomposition of (a) the $Au_{144}(SR)_{60}$ cluster and (b) the Au_{114} core. Color code: Au – magenta (bottom flavor) and dark yellow (middle flavor); S – dark green. The R groups are omitted for clarity.

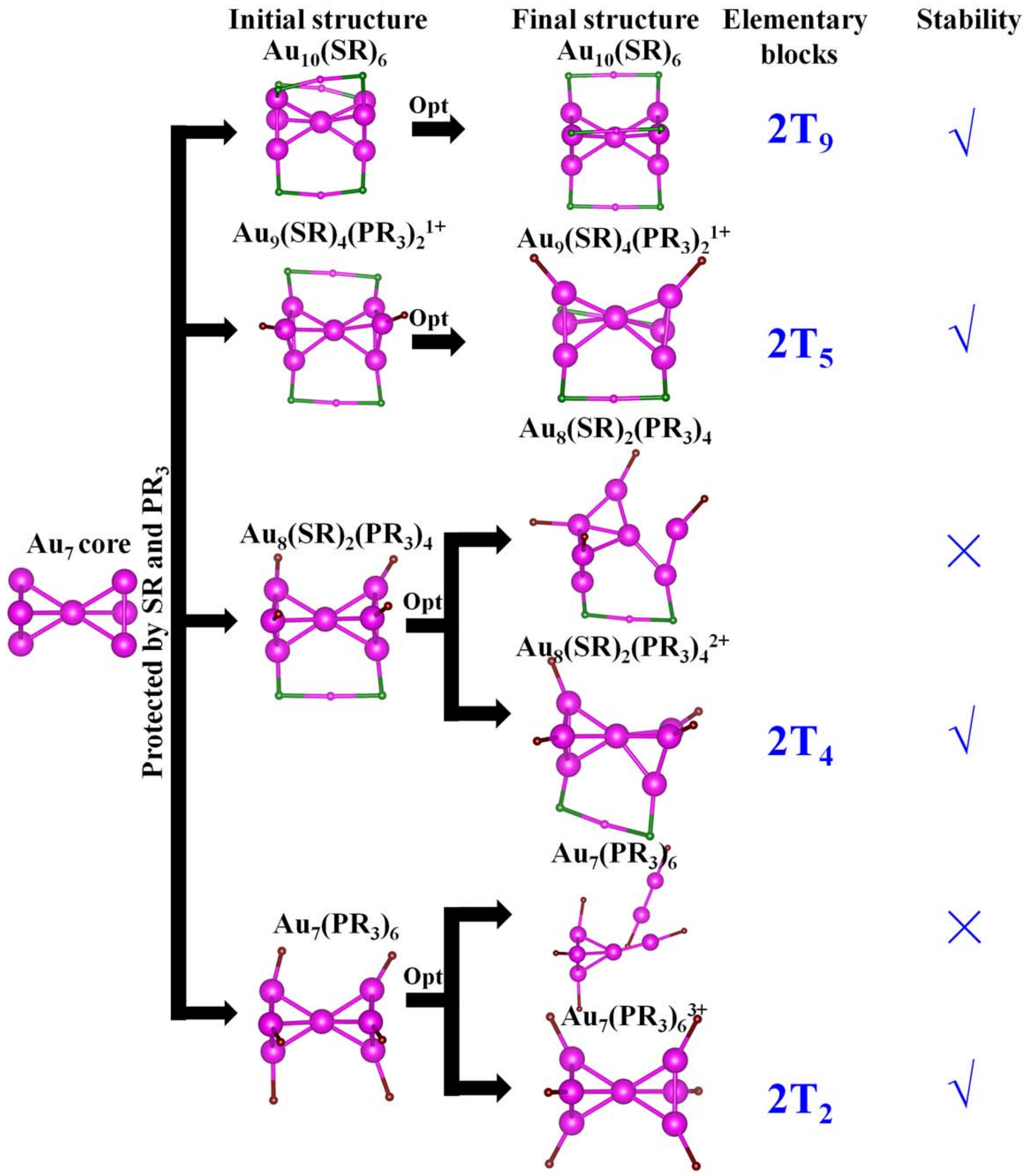

<u>Supplementary</u> Figure 12. The Au_7 core protected by various ligands to form ligand-protected gold clusters. √ and × denote the stable and unstable structures, respectively. Color code: Au – magenta; S – dark green; P – wine. The R groups are omitted for clarity.

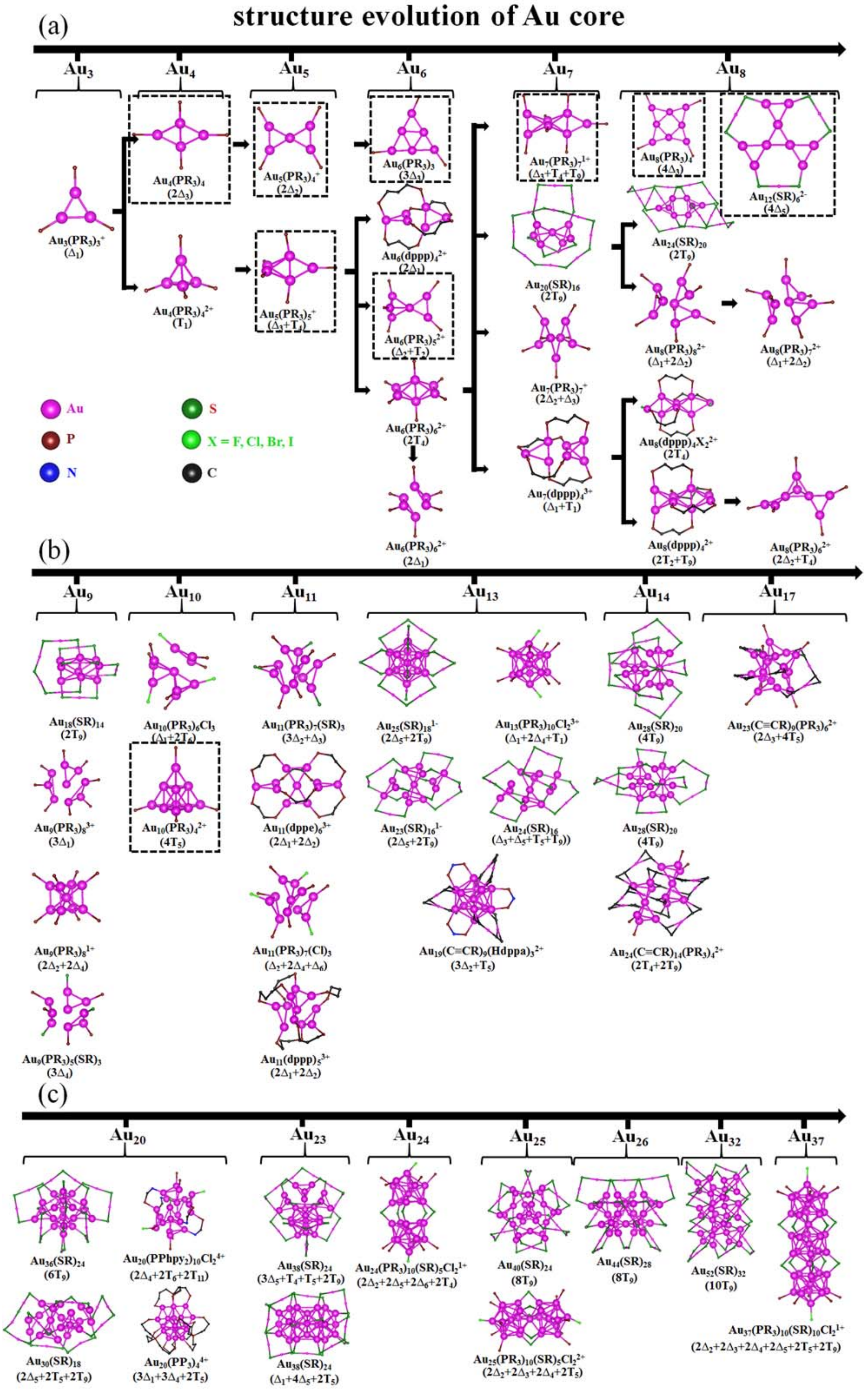

(a)
structure evolution of Au core
Au_3
Au_4
Au_5
Au_6
Au_7
Au_8
$Au_3(PR_3)_3^+$ (Δ_1)
$Au_4(PR_3)_4$ ($2\Delta_3$)
$Au_4(PR_3)_4^{2+}$ (T_1)
$Au_5(PR_3)_4^+$ ($2\Delta_2$)
$Au_5(PR_3)_5^+$ (Δ_3+T_1)
$Au_6(PR_3)_3$ ($3\Delta_3$)
$Au_6(dppp)_4^{2+}$ ($2\Delta_1$)
$Au_6(PR_3)_5^{2+}$ (Δ_2+T_2)
$Au_6(PR_3)_6^{2+}$ ($2T_4$)
$Au_6(PR_3)_6^{2+}$ ($2\Delta_1$)
$Au_7(PR_3)_7^{1+}$ ($\Delta_3+T_4+T_9$)
$Au_{20}(SR)_{16}$ ($2T_9$)
$Au_7(PR_3)_7^+$ ($2\Delta_2+\Delta_3$)
$Au_7(dppp)_4^{3+}$ (Δ_1+T_1)
$Au_8(PR_3)_4$ ($4\Delta_3$)
$Au_{12}(SR)_6^{2-}$ ($4\Delta_5$)
$Au_{24}(SR)_{20}$ ($2T_9$)
$Au_8(PR_3)_8^{2+}$ ($\Delta_1+2\Delta_2$)
$Au_8(PR_3)_7^{2+}$ ($\Delta_1+2\Delta_2$)
$Au_8(dppp)_4X_2^{2+}$ ($2T_4$)
$Au_8(dppp)_4^{2+}$ ($2T_2+T_9$)
$Au_8(PR_3)_6^{2+}$ ($2\Delta_2+T_4$)
Au
P
N
S
X = F, Cl, Br, I
C
(b)
Au_9
Au_{10}
Au_{11}
Au_{13}
Au_{14}
Au_{17}
$Au_{18}(SR)_{14}$ ($2T_9$)
$Au_9(PR_3)_8^{3+}$ ($3\Delta_1$)
$Au_9(PR_3)_8^{1+}$ ($2\Delta_2+2\Delta_4$)
$Au_9(PR_3)_5(SR)_3$ ($3\Delta_4$)
$Au_{10}(PR_3)_6Cl_3$ (Δ_1+2T_6)
$Au_{10}(PR_3)_4^{2+}$ ($4T_5$)
$Au_{11}(PR_3)_7(SR)_3$ ($3\Delta_2+\Delta_3$)
$Au_{11}(dppe)_6^{3+}$ ($2\Delta_1+2\Delta_2$)
$Au_{11}(PR_3)_7(Cl)_3$ ($\Delta_2+2\Delta_4+\Delta_6$)
$Au_{11}(dppp)_5^{3+}$ ($2\Delta_1+2\Delta_2$)
$Au_{25}(SR)_{18}^{1-}$ ($2\Delta_5+2T_9$)
$Au_{13}(PR_3)_{10}Cl_2^{3+}$ ($\Delta_1+2\Delta_4+T_1$)
$Au_{23}(SR)_{16}^{1-}$ ($2\Delta_5+2T_9$)
$Au_{24}(SR)_{16}$ ($\Delta_3+\Delta_5+T_5+T_9$))
$Au_{19}(C{\equiv}CR)_9(Hdppa)_3^{2+}$ ($3\Delta_2+T_5$)
$Au_{28}(SR)_{20}$ ($4T_9$)
$Au_{28}(SR)_{20}$ ($4T_9$)
$Au_{24}(C{\equiv}CR)_{14}(PR_3)_4^{2+}$ ($2T_4+2T_9$)
$Au_{23}(C{\equiv}CR)_9(PR_3)_6^{2+}$ ($2\Delta_3+4T_5$)
(c)
Au_{20}
Au_{23}
Au_{24}
Au_{25}
Au_{26}
Au_{32}
Au_{37}
$Au_{36}(SR)_{24}$ ($6T_9$)
$Au_{20}(PPhpy_2)_{10}Cl_2^{4+}$ ($2\Delta_4+2T_6+2T_{11}$)
$Au_{30}(SR)_{18}$ ($2\Delta_5+2T_5+2T_9$)
$Au_{20}(PP_3)_4^{4+}$ ($3\Delta_1+3\Delta_4+2T_5$)
$Au_{38}(SR)_{24}$ ($3\Delta_5+T_4+T_5+2T_9$)
$Au_{38}(SR)_{24}$ ($\Delta_1+4\Delta_5+2T_5$)
$Au_{24}(PR_3)_{10}(SR)_5Cl_2^{1+}$ ($2\Delta_2+2\Delta_5+2\Delta_6+2T_4$)
$Au_{40}(SR)_{24}$ ($8T_9$)
$Au_{25}(PR_3)_{10}(SR)_5Cl_2^{2+}$ ($2\Delta_2+2\Delta_3+2\Delta_4+2T_5$)
$Au_{44}(SR)_{28}$ ($8T_9$)
$Au_{52}(SR)_{32}$ ($10T_9$)
$Au_{37}(PR_3)_{10}(SR)_{10}Cl_2^{1+}$ ($2\Delta_2+2\Delta_3+2\Delta_4+2\Delta_5+2T_5+2T_9$)

Supplementary Figure 13. Structure evolution of the Au cores (large magenta balls) with increasing number of Au atoms. Color code: Au – magenta; S – dark green; X – light green; P – wine; C – black; N – blue. The R groups are omitted for clarity. The dotted squares denote the newly predicted structures. Others are crystallized structures.

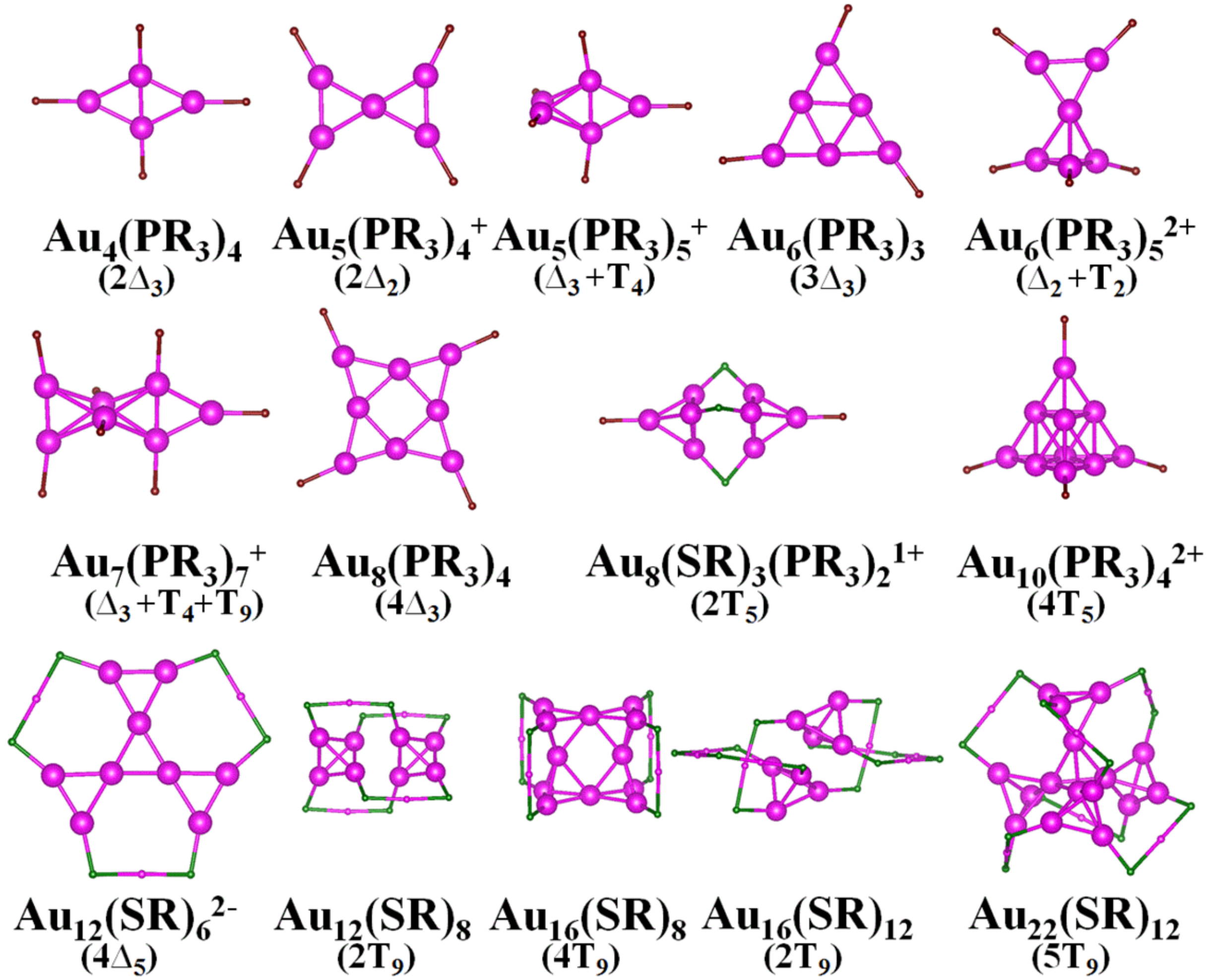

Supplementary Figure 14. Optimized ligand-protected gold clusters whose structures are predicted based on packing various number of elementary blocks. Color code: Au – magenta; S – dark green; P – wine. The R groups are omitted for clarity.

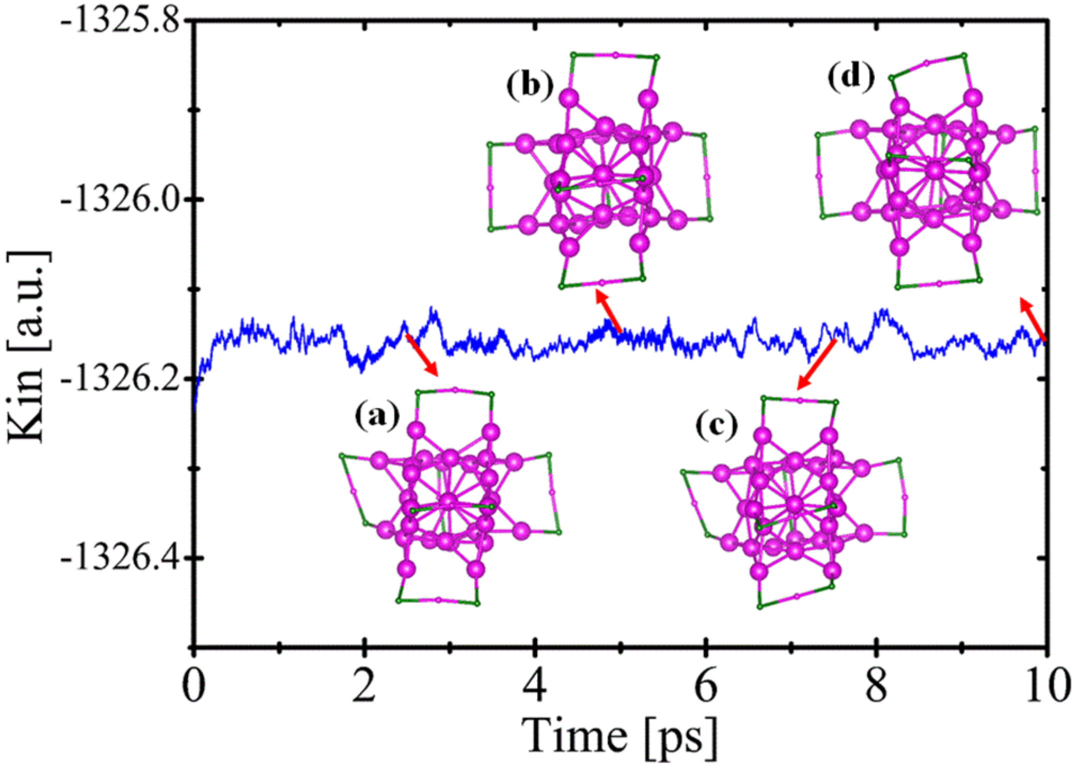

Supplementary Figure 15. Computed kinetic energy (Kin) *vs* simulation time in an *ab initial* molecular dynamics simulation of the $Au_{36}(SH)_{12}$ cluster at 355 K. (a), (b), (c), and (d) correspond to the structure at 2.5, 5, 7.5, and 10 ps, respectively. Color code: Au – magenta; S – dark green. The H atoms are omitted for clarity.

Supplementary Tables:

Supplementary Table 1. Sixteen crystallized structures with strong electron shell closures. In particular, for structures with compact and symmetric (or spherical-like) core, their "superatomic electronic configurations" are given in column 4.

Structures	Morphology of Au core	Number of valence electrons	Superatomic electronic configuration	Elementary blocks at various valence states
$[Au_3(PR_3)_3]^{1+}$ [1]	Spherical	2	$1S^2$	Δ_1
$[Au_4(PR_3)_4]^{2+}$ [2]	Spherical	2	$1S^2$	T_1
$[Au_9(PR_3)_8]^{1+}$ [15]	Spherical	8	$1S^21P^6$	$2\Delta_2+2\Delta_4$
$Au_{11}(PR_3)_7(SR)_3$ [17]	Spherical	8	$1S^21P^6$	$3\Delta_2+\Delta_3$
$[Au_{11}(dppe)_5]^{3+}$ [18]	Spherical	8	$1S^21P^6$	$2\Delta_1+2\Delta_2$

$[Au_{11}(dppe)_6]^{3+}$ [19]	Quasi-planar	8		$2\Delta_1+2\Delta_2$
$Au_{11}(PR_3)_7Cl_3$ [20]	Spherical	8	$1S^21P^6$	$\Delta_2+2\Delta_4+\Delta_6$
$[Au_{11}(PR_3)_8Cl_2]^{1+}$ [21]	Spherical	8	$1S^21P^6$	$\Delta_1+\Delta_2+\Delta_4+\Delta_6$
$[Au_{19}(C\equiv CR)_9(Hdppa)_3]^{2+}$[26]	Spherical	8	$1S^21P^6$	$3\Delta_2+T_5$
$Au_{23}(SR)_{16}^-$ [32]	Disk-like	8		$2\Delta_5+2T_9$
$[Au_{24}(C\equiv CR)_{14}(PR_3)_4]^{2+}$[33]	Rod-like	8		$2T_4+2T_9$
$Au_{24}(SR)_{16}$ [34]	Disk-like	8		$\Delta_3+\Delta_5+T_5+T_9$
$Au_{25}(SR)_{18}^{1-}$ [38, 39]	Spherical	8	$1S^21P^6$	$2\Delta_5+2T_9$
$Au_{28}(SR)_{20}$ [40, 41]	Disk-like	8		$4T_9$
$Au_{44}(SR)_{26}$ [52]	Rod-like	18		$\Delta_1+2\Delta_3+2\Delta_5+4T_9$
$Au_{102}(SR)_{44}$ [55]	Spherical	58	$1S^21P^61D^{10}$ $2S^21F^{14}$ $2P^61G^{18}$	$5\Delta_1+2\Delta_3+16\Delta_5$ $+3T_4+3T_9$

Supplementary Table 2. Total 71 crystallized and predicted (*) structures whose Au cores are composed of the elementary blocks at various valence states.

Structures	Elementary blocks at various valence states	Structures	Elementary blocks at various valence states
$[Au_3(PR_3)_3]^{1+}$ [1]	Δ_1	$Au_{25}(SR)_{18}^{1-}$ [38, 39]	$2\Delta_5+2T_9$
$[Au_4(PR_3)_4]^{2+}$ [2]	T_1	$Au_{28}(SR)_{20}$ [40, 41]	$4T_9$
$[Au_6(PR_3)_6]^{2+}$ [3]	$2\Delta_1$	$Au_{30}(SR)_{18}$ [42]	$2\Delta_5+2T_5+2T_9$
$[Au_6(PR_3)_6]^{2+}$ [4]	$2T_4$	$Au_{30}(SR)_{18}$ [43]	$6T_9$
$[Au_6(dppp)_4]^{2+}$ [5]	$2\Delta_1$	$Au_{30}S(SR)_{18}$ [44]	$\Delta_5+T_5+3T_9$
$[Au_7(PR_3)_7]^{1+}$ [6]	$2\Delta_1+\Delta_3$	$Au_{36}(SR)_{24}$ [45]	$6T_9$
$[Au_7(dppp)_4]^{3+}$ [7]	Δ_1+T_1	$Au_{36}(SR)_8Cl_{20}$ [46]	$2T_5+2T_{10}$
$[Au_8(dppp)_4]^{2+}$ [8]	$2T_4+T_9$	$[Au_{37}(PR_3)_{10}(SR)_{10}Cl_2]^{1+}$ [47]	$2\Delta_2+2\Delta_3+2\Delta_4+$ $2\Delta_5+2T_5+2T_9$
$[Au_8(dppp)_4Cl_2]^{2+}$ [9]	$2T_4$	$Au_{38}S_2(SR)_{20}$ [48]	$2\Delta_5$ $+2T_5+T_8+2T_9$
$[Au_8(PR_3)_8]^{2+}$ [10]	$\Delta_1+2\Delta_2$	$Au_{38}(SR)_{24}$ [49]	$\Delta_1+2\Delta_5+4T_9$
$[Au_8(PR_3)_7]^{2+}$ [11]	$\Delta_1+2\Delta_2$	$Au_{38}(SR)_{24}$ [50]	$3\Delta_5+T_4+T_5+2T_9$
$[Au_8(PR_3)_6]^{2+}$ [12]	$2\Delta_2+T_4$	$Au_{40}(SR)_{24}$ [51]	$8T_9$

$[Au_9(PR_3)_8]^{3+}$ [13]	$3\Delta_1$	$Au_{44}(SR)_{26}$ [52]	$\Delta_1+2\Delta_3+2\Delta_5+4T_9$
$[Au_9(PR_3)_5(SR)_3]$ [14]	$3\Delta_2$	$Au_{44}(SR)_{28}$ [53]	$8T_9$
$[Au_9(PR_3)_8]^{1+}$ [15]	$2\Delta_2+2\Delta_4$	$Au_{52}(SR)_{32}$ [51]	$10T_9$
$[Au_{10}(PR_3)_6Cl_3]^{1+}$ [16]	Δ_4+2T_6	$Au_{92}(SR)_{44}$ [54]	$24T_9$
$Au_{11}(PR_3)_7(SR)_3$ [17]	$3\Delta_2+\Delta_3$	$Au_{102}(SR)_{44}$ [55]	$5\Delta_1+2\Delta_3+16\Delta_5$ $+3T_4+3T_9$
$[Au_{11}(dppe)_5]^{3+}$ [18]	$2\Delta_1+2\Delta_2$	$Au_{130}(SR)_{50}$ [56]	$5\Delta_3+11\Delta_5$ $+4T_4+3T_5+17T_9$
$[Au_{11}(dppe)_6]^{3+}$ [19]	$2\Delta_1+2\Delta_2$	$Au_8(SR)_6$ [57]*	T_9
$Au_{11}(PR_3)_7Cl_3$ [20]	$\Delta_2+2\Delta_4+\Delta_6$	$Au_9(SR)_5$ [57]*	T_9
$[Au_{11}(PR_3)_8Cl_2]^{1+}$ [21]	$\Delta_1+\Delta_2+\Delta_4+\Delta_6$	$Au_{10}(SR)_6$ [57]*	$2T_9$
$[Au_{13}(PR_3)_{10}Cl_2]^{3+}$ [22]	$\Delta_1+2\Delta_4+T_1$	$Au_{10}(SR)_8$ [57]*	T_9
$Au_{14}(PR_3)_8(NO_3)_4$ [23]	$2\Delta_1+\Delta_6+T_{11}+T_{13}$	$Au_{11}(SR)_9$ [57]*	T_9
$Au_{18}(SR)_{14}$ [24, 25]	$2T_9$	$Au_{15}(SR)_{13}$ [58]*	T_9
$[Au_{19}(C{\equiv}CR)_9(Hdppa)_3]^{2+}$[26]	$3\Delta_2+T_5$	$Au_{18}(SR)_{12}$ [59]*	$3T_9$
$[Au_{20}(PPhpy_2)_{10}Cl_2]^{4+}$ [27]	$2\Delta_4+2\Delta_6+2T_{11}$	$Au_{20}(SR)_{12}$ [59]*	$4T_9$
$Au_{20}(PP_3)_4Cl_4$ [28]	$3\Delta_4+3\Delta_6+2T_5$	$Au_{20}(SR)_{16}$ [60]*	$2T_9$
$Au_{20}(SR)_{16}$ [29]	$2T_9$	$Au_{22}(SR)_{18}$ [61]*	$2T_9$
$Au_{21}(SR)_{15}$ [30]	$2\Delta_5+T_4$	$Au_{30}S_2(SR)_{18}$ [62]*	$4T_9$
$[Au_{23}(C{\equiv}CR)_9(PR_3)_6]^{2+}$ [31]	$2\Delta_3+4T_5$	$Au_{40}(SR)_{24}$ [63]*	$6\Delta_5+2T_2$
$Au_{23}(SR)_{16}^{-}$ [32]	$2\Delta_5+2T_9$	$Au_{60}(SR)_{36}$ [64]*	$12T_9$
$[Au_{24}(C{\equiv}CR)_{14}(PR_3)_4]^{2+}$[33]	$2T_4+2T_9$	$Au_{68}(SR)_{34}$ [65]*	$2\Delta_1+\Delta_3+10\Delta_5$ $+T_2+3T_5$
$Au_{24}(SR)_{16}$ [34]	$\Delta_3+\Delta_5+T_5+T_9$	$Au_{68}(SR)_{40}$ [64]*	$14T_9$
$Au_{24}(SR)_{20}$ [35]	$2T_9$	$Au_{76}(SR)_{44}$ [64]*	$16T_9$
$[Au_{24}(PR_3)_{10}(SR)_5X_2]^{1+}$ [36]	$2\Delta_2+2\Delta_5+2\Delta_6+2T_4$	$Au_{144}(SR)_{60}$ [66]*	$20\Delta_5$ $+13T_4+3T_5+6T_9$
$[Au_{25}(PR_3)_{10}(SR)_5Cl_2]^{2+}$ [37]	$2\Delta_2+2\Delta_3+2\Delta_4+2T_5$		

Supplementary Table 3. **The Computed formation energies ΔE_f and HOMO-LUMO gaps of five isoelectronic species.** The formation energies ΔE_f are based on the formula: $Au_2(2e) + n \times Au(0e) = Au_{2+n}(2e)$ (n = 1, 2, 3, and 4)]. The five isoelectronic species $Au_2(2e)$, $Au_3(2e)$, $Au_4(2e)$, $Au_5(2e)$, and $Au_6(2e)$ all have the strong electron shell closures (or the "superatomic orbital" $1S^2$, according to SAC model). The structural optimization of these clusters are performed at three levels of theory: M06/SDD, M06/Aug-cc-VTZ-PP (M06/AVTZ-PP), and M06/Aug-cc-DTZ-PP (M06/AVDZ-PP), respectively.

	$Au_2(2e)$	$Au_3(2e)$ or Au_3^{+}	$Au_4(2e)$ or Au_4^{2+}	$Au_5(2e)$ or Au_5^{3+}		$Au_6(2e)$ or Au_6^{4+}	
	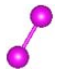	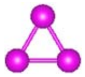	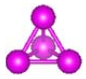	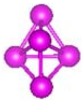	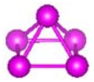	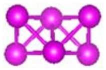	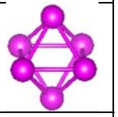
ΔE_f /eV (M06/SDD)	0	**-3.30**	**-1.99**	3.72	unstable	unstable	12.88
ΔE_f /eV (M06/AVTZ-PP)	0	**-3.36**	**-2.10**	3.57	3.68	12.67	12.64
ΔE_f /eV (M06/AVQZ-PP)	0	**-3.36**	**-2.09**	3.60	3.72	12.74	12.71
HOMO-LUMO gap/eV (M06/SDD)	3.54	**4.63**	**5.17**	4.14	unstable	unstable	4.49

Supplementary Table 4. The identified elementary blocks and their corresponding valence states, and the HOMO-LUMO (H-L) gaps of the newly predicted ligand-protected gold clusters whose Au cores are built based on the elementary block packing model. The R group is simplified by H atom.

Structures	Elementary blocks at various valence states	H-L gap/eV	Structures	Elementary blocks at various valence states	H-L gap/eV
$Au_4(PR_3)_4$	$2\Delta_3$	2.97	$[Au_{10}(PR_3)_4]^{2+}$	$4T_5$	3.48
$[Au_5(PR_3)_4]^{1+}$	$2\Delta_2$	4.00	$[Au_{12}(SR)_6]^{2-}$	$4\Delta_5$	3.16
$[Au_5(PR_3)_5]^{1+}$	Δ_3+T_4	3.56	$Au_{12}(SR)_8$	$2T_9$	3.10
$Au_6(PR_3)_3$	$3\Delta_3$	3.24	$Au_{16}(SR)_8$	$4T_9$	2.86
$[Au_6(PR_3)_5]^{2+}$	Δ_2+T_2	4.03	$Au_{16}(SR)_{12}$	$2T_9$	2.56
$[Au_7(PR_3)_7]^{1+}$	$\Delta_3+T_4+T_9$	2.59	$Au_{22}(SR)_{12}$	$5T_9$	3.46
$Au_8(PR_3)_4$	$4\Delta_3$	2.31	$Au_{36}(SR)_{12}$	$12T_9$	2.20
$[Au_8(PR_3)_2(SR)_3]^{1+}$	$2T_5$	4.54	$Au_{42}(SR)_{14}$	$14T_9$	2.00

Supplementary Methods:

The structural optimizations of the ligand-protected gold clusters were performed using density functional theory (DFT) methods with the the M06 functional [67] and the all-electron basis set 6-31G* for H, C, P, Cl, S, pseudopotential basis set LANL2DZ for Au, as implemented in the Gaussian 09 program package [68]. The –R group is replaced by –H to lower computational cost. Using the DFT method implemented in the CP2K package [69,70], *ab initio* molecular dynamics simulations for $Au_{36}(SH)_{12}$ were performed in the canonical ensemble, with using the Nose-Hoover thermostat and a time step of 1 fs, and at a finite temperature of 355 K. The exchange correlation potential was described by the generalized-gradient approximation (GGA) with the

spin-polarized functional of Perdew-Burke-Ernzerh (PBE) [71]. Wavefunctions were expanded in triple-ζ Gaussian basis sets with an auxiliary plane-wave basis and a cutoff energy of 300 Ry. Core electrons were modeled by scalar relativistic norm-conserving pseudopotentials [72,73] with 11, 6, and 1 valence electrons of Au, S, and H, respectively. Brillouin zone integration was performed with a reciprocal space mesh consisting of only the Γ-point.

Supplementary references:

1. Robilotto, T. J., Bacsa, J., Gray, T. G. & Sadighi, J. P. Synthesis of a trigold monocation: An isolobal analogue of $[H_3]^+$. *Angew. Chem.* **124**, 12243–12246 (2012).
2. Zeller, E., Beruda, H. & Schmidbaur, H. Tetrahedral gold cluster $[Au_4]^{2+}$: Crystal structure of $\{[(^tBu)_3PAu]_4\}^{2+}(BF_4^-)_2\cdot 2CHCl_3$. *Inorg. Chem.* **32**, 3203–3204 (1993).
3. Briant, C. E., Hall, K. P., Mingos, D. M. P. & Wheeler, A. C. Synthesis and structural characterisation of hexakis (triphenyl phosphine)-hexagold (2+) nitrate, $[Au_6(PPh_3)_6][NO_3]_2$, and related clusters with edge-sharing bitetrahedral geometries. *J. Chem. Soc., Dalton Trans.* 687–692 (1986).
4. Bellon, P., Manassero, M. & Sansoni, M. An octahedral gold cluster: crystal and molecular structure of hexakis[tris-(p-tolyl)phosphine]-octahedro-hexagold bis(tetraphenylborate). *J. Chem. Soc., Dalton Trans*. 2423–2427 (1973)
5. Van Der Velden, J. W. A., Bour, J. J., Steggerda, J. J., Beurskens, P. T., Roseboom, M. & Noordik, J. H. Gold clusters. Tetrakis[1,3-bis(diphenylphosphino)propane]hexagold dinitrate: preparation, x-ray analysis, and gold-197 Moessbauer and phosphorus-31{proton} NMR spectra. *Inorg. Chem.* **21**, 4321–4324 (1982).
6. Van Der Velden, J. W. A., Beurskens, P. T., Bour, J. J., Bosman, W. P., Noordik, J. H., Kolenbrander, M. & Buskes, J. A. K. M. Intermediates in the formation of gold clusters. Preparation and x-ray analysis of $[Au_7(PPh_3)_7]^+$ and synthesis and characterization of $[Au_8(PPh_3)_6I]PF_6$. *Inorg. Chem.* **23**, 146–151 (1984).
7. Shichibu, Y., Zhang, M., Kamei, Y. & Konishi, K. $[Au_7]^{3+}$: A missing link in the four-electron gold cluster family. *J. Am. Chem. Soc.* **136**, 12892−12895 (2014).
8. Kobayashi, N., Kamei, Y., Shichibu, Y. & Konishi, K. Protonation-induced chromism of pyridylethynyl-appended [core+exo]-type Au_8 clusters. Resonance-coupled electronic perturbation through π-conjugated group. *J. Am. Chem. Soc.* **135**, 16078–16081 (2013).
9. Kamei, Y., Shichibu, Y. & Konishi, K. Generation of small gold clusters with unique geometries through cluster-to-cluster transformations: Octanuclear clusters with edgesharing gold tetrahedron motifs. *Angew. Chem. Int. Ed.* **50**, 7442–7445 (2011).
10. Manassero, M., Naldini, L. & Sansoni, M. A new class of gold cluster compounds. Synthesis and *X*-ray structure of the octakis(triphenylphosphinegold) dializarinsulphonate, $[Au_8(PPh_3)_8](aliz)_2$. *J. Chem. Soc., Chem. Commun.* 385–386 (1979).
11. Van Der Velden, J. W. A., Bour, J. J., Bosman, W. P. & Noordik, J. H. Synthesis and *X*-ray crystal structure determination of the cationic gold cluster compound $[Au_8(PPh_3)_7](NO_3)_2$. *J. Chem. Soc., Chem. Commun.* 1218–1219 (1981).
12. Yang, Y. & Sharp, P. R. New gold clusters $[Au_8L_6](BF_4)_2$ and $[(AuL)_4](BF_4)_2$ (L = $P(mesityl)_3$). *J. Am. Chem. Soc.* **116**, 6983–6984 (1994).
13. Bellon, P. L., Cariati, F., Manassero, M., Naldini, L. & Sansoni, M. Novel gold clusters. Preparation, properties, and X-ray structure determination of salts of octakis(triarylphosphine)enneagold, $[Au_9L_8]X_3$. *J. Chem. Soc. D* 1423–1424 (1971).

14. Cooper, M. K., Dennis, G. R., Henrick, K. & Mcpartlin, M. A new type of gold cluster compound. The syntheses and X-ray structure analysis of pentakis (tricyclohexylphosphino) tris (thiocyanato) enneagold, $[Au_9\{P(C_6H_{11})_3\}_5(SCN)_3]$, and bis {tri (cyclohexyl) phosphinato} gold (I) hexafluorophosphate, $[Au\{P(C_6H_{11})_3\}_2][PF_6]$. *Inorg. Chim. Acta.* **45**, L151–L152 (1980).
15. Van Der Velden, J. W. A., Bour, J. J., Bosman, W. P., Noordik, J. H. & Beurskens, P. T. The electrochemical preparation of $[Au_9(PPh_3)_8]^+$. A comparative study of the structures and properties of $[Au_9(PPh_3)_8]^+$ and $[Au_9(PPh_3)_8]^{3+}$. *Recl. Trav. Chim. Pays-Bas* **103**, 13–16 (1984).
16. Briant, C. E., Hall, K. P., Wheeler, A. C. & Mingos, D. M. P. Structural characterisation of $[Au_{10}Cl_3(PCy_2Ph)_6](NO_3)$(Cy = cyclohexyl) and the development of a structural principle for high nuclearity gold clusters. *J. Chem. Soc., Chem. Commun.* 248-250 (1984).
17. Nunokawa, K., Onakaa, S., Itoa, M., Horibea, M., Yonezawab, T., Nishiharab, H., Ozekic, T., Chibac, H., Watased, S. & Nakamoto, M. Synthesis, single crystal X-ray analysis, and TEM for a single-sized Au_{11} cluster stabilized by SR ligands: The interface between molecules and particles. *J. Organomet. Chem.* **691**, 638–642 (2006).
18. Shichibu, Y., Kamei, Y. & Konishi, K. Unique [core+two] structure and optical property of a dodeca-ligated undecagold cluster: critical contribution of the exo gold atoms to the electronic structure. *Chem. Commun.* **48**, 7559–7561 (2012).
19. Smits, J. M. M., Bour, J. J., Vollenbroek, F. A. & Beurskens, P. T. Preparation and X-ray structure determination of [pentakis{1,3-bis(diphenylphosphino)propane}] undecagoldtris(thiocyanate), $[Au_{11}\{PPh_2C_3H_6PPh_2\}_5](SCN)_3$. *J. Cryst. Spectrosc.* **13**, 355–363 (1983).
20. Bellon, P., Manassero, M. & Sansoni, M. Crystal and molecular structure of tri-iodoheptakis(tri-*p*-fluorophenylphosphine)undecagold. *J. Chem. Soc., Dalton Trans.* 1481–1487 (1972).
21. McKenzie, L. C., Zaikova, T. O. & Hutchison, J. E. Structurally Similar triphenylphosphine-stabilized undecagolds, $Au_{11}(PPh_3)_7Cl_3$ and $[Au_{11}(PPh_3)_8Cl_2]Cl$, exhibit distinct ligand exchange pathways with glutathione. *J. Am. Chem. Soc.* **136**, 13426−13435 (2014).
22. Briant, C. E., Tobald, B. R. C., White, J. W., Bell, L. K. & Mingos, D. M. P. Synthesis and X-ray structural characterization of the centered icosahedral gold cluster compound $[Au_{13}(PMe_2Ph)_{10}Cl_2](PF_6)_3$; the realization of a theoretical prediction. *J. Chem. Soc. Chem. Commun.* **5**, 201–202 (1981).
23. Gutrath, B. S., Oppel, I. M., Presly, O., Beljakov, I., Meded, V., Wenzel, W. & Simon, U. $[Au_{14}(PPh_3)_8(NO_3)_4]$: An example of a new class of $Au(NO_3)$-Ligated superatom complexes. *Angew. Chem. Int. Ed.* **52**, 3529–3532 (2013).
24. Das, A., Liu, C., Byun, H. Y., Nobusada, K., Zhao, S., Rosi, N. L. & Jin, R. Structure determination of $[Au_{18}(SR)_{14}]$. *Angew. Chem. Int. Ed.* **54**, 3140–3144 (2015).
25. Chen, S., Wang, S., Zhong, J., Song, Y., Zhang, J., Sheng, H., Pei, Y. & Zhu, M. The structure and optical properties of the $[Au_{18}(SR)_{14}]$ nanocluster. *Angew. Chem. Int. Ed.* **54**, 3145–3149 (2015).
26. Wan, X., Tang, Q., Yuan, S., Jiang, D. & Wang, Q. M. Au_{19} nanocluster featuring a V−shaped alkynyl−gold motif. *J. Am. Chem. Soc.* **137**, 652−655 (2015).
27. Wan, X., Tang, Q., Yuan, S., Jiang, D. & Wang, Q. M. Au_{20} nanocluster protected by hemilabile phosphines. *J. Am. Chem. Soc.* **134**, 14750−14752 (2012).
28. Wan, X., Yuan, S., Lin, Z. & Wang, Q. M. A chiral gold nanocluster Au_{20} protected by

tetradentate phosphine ligands. *Angew. Chem. Int. Ed.* **53**, 2923–2926 (2014).
29. Zeng, C., Liu, C., Chen, Y., Rosi, N. L. & Jin, R. Gold-thiolate ring as a protecting motif in the $Au_{20}(SR)_{16}$ nanocluster and implications. *J. Am. Chem. Soc.* **136**, 11922–11925 (2014).
30. Chen, S., Xiong, L., Wang, S., Ma, Z., Jin, S., Sheng, H., Pei, Y. & Zhu, M. Total structure determination of $Au_{21}(S\text{-}Adm)_{15}$ and geometrical/electronic structure evolution of thiolated gold nanoclusters. *J. Am. Chem. Soc.* **138**, 10754–10757 (2016).
31. Wan, X., Yuan, S., Tang, Q., Jiang, D. & Wang, Q. M. Alkynyl-protected Au_{23} nanocluster: A 12-electron system. *Angew. Chem. Int. Ed.* **54**, 5977–5980 (2015).
32. Das, A., Li, T., Nobusada, K., Zeng, C., Rosi, N. L. & Jin, R. Nonsuperatomic $[Au_{23}(SC_6H_{11})_{16}]^-$ nanocluster featuring bipyramidal Au_{15} kernel and trimeric $Au_3(SR)_4$ motif. *J. Am. Chem. Soc.* **135**, 18264–18267 (2013).
33. Wan, X., Xu, W. W., Yuan, S., Gao, Y., Zeng, X. C. & Wang, Q. M. A near-infrared-emissive alkynyl-protected Au_{24} nanocluster. *Angew. Chem. Int. Ed.* **54**, 9683–9686 (2015).
34. Crasto, D., Barcaro, G., Stener, M., Sementa, L., Fortunelli, A. & Dass, A. $Au_{24}(Sadm)_{16}$ nanomolecules: X-ray crystal structure, theoretical analysis, adaptability of adamantane ligands to form $Au_{23}(Sadm)_{16}$ and $Au_{25}(Sadm)_{16}$, and its relation to $Au_{25}(SR)_{18}$. *J. Am. Chem. Soc.* **136**, 14933–14940 (2014).
35. Das, A., Li, T., Li, G., Nobusada, K., Zeng, C., Rosi, N. L. & Jin, R. Crystal structure and electronic properties of a thiolate-protected Au_{24} nanocluster. *Nanoscale* **6**, 6458–6462 (2014).
36. Das, A., Li, T., Nobusada, K., Zeng, Q., Rosi, N. L. & Jin, R. Total structure and optical properties of a phosphine/thiolate-protected Au_{24} nanocluster. *J. Am. Chem. Soc.* **134**, 20286−20289 (2012).
37. Shichibu, Y., Negishi, Y., Watanabe, T., Chaki, N. K., Kawaguchi, H. & Tsukuda, T. Biicosahedral gold clusters $[Au_{25}(PPh_3)_{10}(SC_nH_{2n+}1)_5Cl_2]^{2+}$ (n = 2–18): A stepping stone to cluster-assembled materials. *J. Phys. Chem. C* **111**, 7845–7847 (2007).
38. Zhu, M., Aikens, C. M., Hollander, F. J., Schatz, G. C. & Jin, R. Correlating the crystal structure of a thiol-protected Au_{25} cluster and optical properties. *J. Am. Chem. Soc.* **130**, 5883–5885 (2008).
39. Heaven, M. W., Dass, A., White, P. S., Holt, K. M. & Murray, R. W. Crystal structure of the gold nanoparticle $[N(C_8H_{17})_4][Au_{25}(SCH_2CH_2Ph)_{18}]$. *J. Am. Chem. Soc.* **130**, 3754–3755 (2008).
40. Zeng, C., Li, T., Das, A., Rosi, N. L. & Jin, R. Chiral structure of thiolate-protected 28-gold-atom nanocluster determined by X-ray crystallography. *J. Am. Chem. Soc.* **135**, 10011–10013 (2013).
41. Chen, Y., Liu, C., Tang, Q., Zeng, C., Higaki, T., Das, A., Jiang, D., Rosi, N. L. & Jin, R. Isomerism in $Au_{28}(SR)_{20}$ nanocluster and stable structures. *J. Am. Chem. Soc.* **138**, 1482–1485 (2016).
42. Dass, A., Jones, T., Rambukwella, M., Crasto, D., Gagnon, K. J., Sementa, L., De Vetta, M., Baseggio, O., Aprà, E., Stener, M. & Fortunelli, A. Crystal structure and theoretical analysis of green gold $Au_{30}(S\text{-}tBu)_{18}$ nanomolecules and their relation to $Au_{30}S(S\text{-}tBu)_{18}$. *J. Phys. Chem. C* **120**, 6256−6261 (2016).
43. Higaki, T., Liu, C., Zeng, C., Jin, R., Chen, Y., Rosi, N. L. & Jin, R. Controlling the atomic structure of Au_{30} nanoclusters by a ligand-based strategy. *Angew. Chem. Int. Ed.* **55**, 6694 – 6697 (2016).

44. Crasto, D., Malola, S., Brosofsky, G., Dass, A. & Häkkinen, H. Single crystal XRD structure and theoretical analysis of the chiral $Au_{30}S(S\text{-}t\text{-}Bu)_{18}$ Cluster. *J. Am. Chem. Soc.* **136**, 5000–5005 (2014).
45. Zeng, C., Qian, H., Li, T., Li, G., Rosi, N. L., Yoon, B., Barnett, R. N., Whetten, R. L., Landman, U. & Jin, R. Total structure and electronic properties of the gold nanocrystal $Au_{36}(SR)_{24}$. *Angew. Chem. Int. Ed.* **51**, 13114–13118 (2012).
46. Yang, S., Chai, J., Song, Y., Kang, X., Sheng, H., Chong, H. & Zhu, M. A new crystal structure of Au_{36} with a Au_{14} kernel cocapped by thiolate and chloride. *J. Am. Chem. Soc.* **137**, 10033−10035 (2015).
47. Jin, R., Liu, C., Zhao, S., Das, A., Xing, H., Gayathri, C., Xing, Y., Rosi, N. L., Gil, R. R. & Jin, R. Tri-icosahedral gold nanocluster $[Au_{37}(PPh_3)_{10}(SC_2H_4Ph)_{10}X_2]^+$: Linear assembly of icosahedral building blocks. *ACS Nano* **9**, 8530–8536 (2015).
48. Liu, C., Li, T., Li, G., Nobusada, K., Zeng, C., Pang, G., Rosi, N. L. & Jin, R. Observation of body-centered cubic gold nanocluster. *Angew. Chem. Int. Ed.* **54**, 9826–9829 (2015).
49. Qian, H., Eckenhoff, W. T., Zhu, Y., Pintauer, T. & Jin, R. Total structure determination of thiolate-protected Au_{38} nanoparticles. *J. Am. Chem. Soc.* **132**, 8280–8281 (2010).
50. Tian, S., Li, Y., Li, M., Yuan, J., Yang, J., Wu, Z. & Jin, R. Structural isomerism in gold nanoparticles revealed by X-ray crystallography. *Nat. Commun.* **6**, 8667, (2015).
51. Zeng, C., Chen, Y., Liu, C., Nobusada, K., Rosi, N. L. & Jin, R. Gold tetrahedra coil up: Kekulé-like and double helical superstructures. *Sci. Adv.* **1**, e1500425 (2015).
52. Liao, L., Zhuang, S., Yao, C., Yan, N., Chen, J., Wang, C., Xia, N., Liu, X., Li, M., Li, L., Bao, X. & Wu, Z. Structure of chiral $Au_{44}(2,4\text{-}DMBT)_{26}$ nanocluster with an 18-electron shell closure. *J. Am. Chem. Soc.***138**, 10425–10428 (2016).
53. Zeng, C., Chen, Y., Iida, K., Nobusada, K., Kirschbaum, K., Lambright, K. J. & Jin, R. Gold quantum boxes: On the periodicities and the quantum confinement in the Au_{28}, Au_{36}, Au_{44}, and Au_{52} magic series. *J. Am. Chem. Soc.* **138**, 3950–3953 (2016).
54. Zeng, C., Liu, C., Chen, Y., Rosi, N. L. & Jin, R. atomic structure of self-assembled monolayer of thiolates on a tetragonal Au_{92} nanocrystal. *J. Am. Chem. Soc.* **138**, 8710–8713 (2016).
55. Jadzinsky, P. D., Calero, G., Ackerson, C. J., Bushnell, D. A. & Kornberg, R. D. Structure of a thiol monolayer-protected gold nanoparticle at 1.1 Å resolution. *Science* **318**, 430–433 (2007).
56. Chen, Y., Zeng, C., Liu, C., Kirschbaum, K., Gayathri, C., Gil, R. R., Rosi, N. L. & Jin, R. Crystal structure of barrel-shaped chiral $Au_{130}(p\text{-}MBT)_{50}$ nanocluster. *J. Am. Chem. Soc.* **137**, 10076–10079 (2015).
57. Liu, C., Pei, Y., Sun, H. & Ma, J. The nucleation and growth mechanism of thiolate-protected Au nanoclusters. *J. Am. Chem. Soc.* **137**, 15809–15816 (2015).
58. Jiang, D., Overbury, S. H. & Dai, S. Structure of $Au_{15}(SR)_{13}$ and its implication for the origin of the nucleus in thiolated gold nanoclusters. *J. Am. Chem. Soc.* **135**, 8786−8789 (2013).
59. Cheng, L., Yuan, Y., Zhang, X. & Yang, J. Superatom networks in thiolate-protected gold nanoparticles. *Angew. Chem. Int. Ed.* **52**, 9035 –9039 (2013).
60. Pei, Y., Gao, Y., Shao, N. & Zeng, X. C. Thiolate-protected $Au_{20}(SR)_{16}$ cluster: Prolate Au_8 core with new $[Au_3(SR)_4]$ staple motif. *J. Am. Chem. Soc.* **131**, 13619–13621 (2009).

61. Pei, Y., Tang, J., Tang, X., Huang, Y. & Zeng, X. C. New structure model of $Au_{22}(SR)_{18}$: Bitetrahederon golden kernel enclosed by [$Au_6(SR)_6$] Au(I) complex. *J. Phys. Chem. Lett.* **6**, 1390−1395 (2015).
62. Tian, Z. & Cheng, L. Electronic and geometric structures of Au_{30} clusters: a network of 2e-superatom Au cores protected by tridentate protecting motifs with u_3-S. *Nanoscale* **8**, 826–834 (2016).
63. Malola, S., Lehtovaara, L., Knoppe, S., Hu, K., Palmer, R. E., Bürgi, T. & Häkkinen, H. $Au_{40}(SR)_{24}$ cluster as a chiral dimer of 8-electron superatoms: Structure and optical properties. *J. Am. Chem. Soc.* **134**, 19560–19563 (2012).
64. Xu, W. W., Li, Y., Gao, Y. & Zeng, X. C. Unraveling a generic growth pattern in structure evolution of thiolate-protected gold nanoclusters. *Nanoscale* **8**, 7396–7401 (2016).
65. Xu, W. W. & Gao, Y. Unraveling the atomic structures of the $Au_{68}(SR)_{34}$ nanoparticles. *J. Phys. Chem. C* **119**, 14224−14229 (2015)
66. Lopez-Acevedo, O., Akola, J., Whetten, R. L., Grönbeck, H. & Häkkinen, H. Structure and bonding in the ubiquitous icosahedral metallic gold cluster $Au_{144}(SR)_{60}$. *J. Phys. Chem. C* **113**, 5035–5038 (2009).
67. Zhao, Y., Truhlar, D. G. The M06 suite of density functionals for main group thermochemistry, thermochemical kinetics, noncovalent interactions, excited states, and transition elements: two new functionals and systematic testing of four M06-class functionals and 12 other functionals. *Theor. Chem. Acc.* **120**, 215–241 (2008).
68. Frisch, M. J., Trucks, G. W., Schlegel, H. B., Scuseria, G. E., Robb, M. A., Cheeseman, J. R., Zakrzewski, V. G., Montgomery, J. A., Stratmann, Jr., R. E., Burant, J. C., Dapprich, S., Millam, J. M., Daniels, A. D., Kudin, K. N., Strain, M. C., Farkas, O., Tomasi, J., Barone, V., Cossi, M., Cammi, R., Mennucci, B., Pomelli, C., Adamo, C., Clifford, S., Ochterski, J., Petersson, G. A., Ayala, P. Y., Cui, Q., Morokuma, K., Malick, D. K., Rabuck, A. D., Raghavachari, K., Foresman, J. B., Cioslowski, J., Ortiz, J. V., Stefanov, B. B., Liu, G., Liashenko, A., Piskorz, P., Komaromi, I., Gomperts, R., Martin, R. L., Fox, D. J., Keith, T., Al-Laham, M. A., Peng, C. Y., Nanayakkara, A., Gonzalez, C., Challacombe, M., Gill, P. M. W., Johnson, B., Chen, W., Wong, M. W., Andres, J. L., Gonzalez, C., Head-Gordon, M., Replogle, E. S. & Pople, J. A. *Gaussian 09, Revision B.01* (Gaussian, Inc., 2010).
69. Lippert, G., Hutter, J. & Parrinello, M., The gaussian and augmented-plane-wave density functional method for *ab initio* molecular dynamics simulations. *Theor. Chem. Acc.* **103**, 124−140 (1999).
70. VandeVondele, J., Krack, M., Mohamed, F., Parrinello, M., Chassaing, T. & Hutter, J. Quickstep: Fast and accurate density functional calculations using a mixed gaussian and plane waves approach. *Comput. Phys. Commun.* **167**, 103−128 (2005).
71. Perdew, J. P., Burke, K. & Ernzerhof, M. Generalized gradient approximation made simple. *Phys. Rev. Lett.* **77**, 3865−3868 (1996).
72. Goedecker, S., Teter, M. & Hutter, J. Separable dual-space gaussian pseudopotentials. *Phys. Rev. B* **54**, 1703−1710 (1996).
73. Krack, M. Pseudopotentials for H to Kr optimized for gradient-corrected exchange-correlation functionals. *Theor. Chem. Acc.* **114**, 145−152 (2005).
